# Development of a VEGF-activated scaffold with enhanced angiogenic and neurogenic properties for chronic wound healing applications†

**DOI:** 10.1039/d4bm01051e

**Published:** 2025-02-14

**Authors:** Juan Carlos Palomeque Chávez, Matthew McGrath, Cian O'Connor, Adrian Dervan, James E. Dixon, Cathal J. Kearney, Shane Browne, Fergal J. O'Brien

**Affiliations:** a Tissue Engineering Research Group, Department of Anatomy & Regenerative Medicine, Royal College of Surgeons in Ireland Dublin Ireland fjobrien@rcsi.ie +353-1-4022149; b Advanced Materials and Bioengineering Research Centre (AMBER), Royal College of Surgeons in Ireland and Trinity College Dublin Dublin Ireland; c Kearney Lab, Department of Biomedical Engineering, University of Massachusetts Armhest USA; d Trinity Centre for Biomedical Engineering, Trinity College Dublin Dublin Ireland; e Regenerative Medicine & Cellular Therapies (RMCT), Biodiscovery Institute (BDI), School of Pharmacy, University of Nottingham Nottingham UK; f NIHR Nottingham Biomedical Research Centre, University of Nottingham Nottingham UK; g Centre for Research in Medical Devices (CÚRAM), University of Galway Galway Ireland

## Abstract

Chronic wounds remain in a state of disrupted healing, impeding neurite outgrowth from injured nerves and poor development of new blood vessels by angiogenesis. Current therapeutic approaches primarily focus on the restoration of vascularization and overlook the need of nerve regeneration for complete healing. Vascular endothelial growth factor (VEGF) is a critical growth factor supporting angiogenesis in wound healing, promoting vascularization and has also demonstrated neuro-protective capabilities in both central and peripheral nervous system. While the delivery of pro-regenerative recombinant growth factors has shown promise, gene delivery offers greater stability, reduced off-target side effects, diminished cytotoxicity, and lower production costs. In this context, the overarching goal of this study was to develop a VEGF-activated scaffold with the potential to provide a multifaceted response that enhances both angiogenesis and nerve repair in wound healing through the localized delivery of plasmid encoding VEGF (*p*VEGF) encapsulated within the GET peptide system. Initially, delivery of *p*VEGF/GET nanoparticles to dermal fibroblasts led to higher VEGF protein expression without a compromise in cell viability. Transfection of dermal fibroblasts and endothelial cells on the VEGF-activated scaffolds resulted in enhanced VEGF expression, improved endothelial cell migration and organization into vascular-like structures. Finally, the VEGF-activated scaffolds consistently displayed enhanced neurogenic ability through improved neurite outgrowth from neural cells in *in vitro* and *ex vivo* models. Taken together, the VEGF-activated scaffold demonstrates multifaceted outcomes through the induction of pro-angiogenic and neurogenic responses from dermal, vascular and neural cells, illustrating the potential of this platform for the healing of chronic wounds.

## Abstract 2

Schematic showing the development of the multifaceted VEGF-activated scaffolds to promote pro-regenerative environments for chronic wound applications.

## Graphical abstract

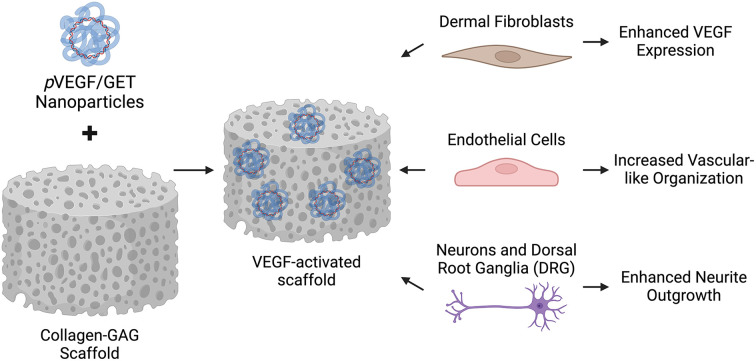

## 1. Introduction

Chronic wounds are characterized by their failure to progress through the normal wound healing process in a sequential and timely manner, significantly increasing the risk of complications.^1^ Upon injury, healthy skin naturally progresses through four overlapping and sequential healing stages, namely: hemostasis, inflammation, proliferation and remodeling.^2^ However, chronic wounds remain in a state of prolonged inflammation, hampering healing, impeding neurite outgrowth from injured nerves and hindering angiogenesis.^3,4^ Importantly, an absence of proper angiogenesis leads to a shortage of essential nutrients and oxygen to the affected area, further aggravating the damage in the tissue.^5^ Similarly, while its impact is often overlooked, insufficient nerve regeneration (neurogenesis) exacerbates vascular dysfunction, inhibits the recovery of motor and sensory functions, and deprives the area from the secretion of factors that promote regeneration.^6–9^

Approximately 1–2% of individuals in developed countries experience a chronic wound during their lifetime,^10^ underlining the importance of proper treatment. However, current chronic wound treatments are primarily reactive, suboptimal and non-viable in many cases due to their inability to deal with the underlying pathology,^11–13^ providing limited benefit and repair. Vascular dysfunction is often addressed through debridement of the wound and optimization of blood flow to the affected area,^3,14,15^while nerve damage is typically managed through surgical intervention, medication, physical therapy and pain management treatment plans.^16^ Moreover, the recovery of angiogenesis is often favored over nerve regeneration despite the synergistic pro-regenerative activity of both processes within the injury site.^6,7^ Clinical approaches to overcome the limitations of traditional treatments include the use of extracellular matrix (ECM)-derived and synthetic biomaterials, such as the Integra®, Dermagraft®, Apligraft®, Alloderm® and Novosorb® matrices.^17–21^ These have been designed to mimic the native structure of skin, serving as templates for wound healing in chronic wounds.^22^ However, these products often fall short in addressing the challenges of delayed angiogenesis and impaired nerve repair, highlighting the need for advanced biomaterials capable of addressing the underlying pathology to promote regeneration.^23–25^

Angiogenesis is a well-known process that involves complex signaling among multiple cells, such as fibroblasts and endothelial cells, facilitated by the activity of various growth factors, including vascular endothelial growth factor (VEGF).^26^ VEGF is critical to supporting angiogenesis in wound healing, promoting vascularization and wound repair.^27^ Apart from its pro-angiogenic effect, VEGF has also been linked with neuro-protective capabilities in both central,^28^ and peripheral nervous system.^29^ Therefore, VEGF might provide a neurogenic stimulus in addition to its widely established pro-angiogenic effect to elicit a multifaceted therapeutic response for the treatment of chronic wounds.

Exogenous recombinant VEGF growth factor delivery has been explored to promote vascularization,^30^ but these growth factor-based therapies have failed to deliver due to their susceptibility to deactivation, low tissue permeation,^31^ and inconsistent clinical outcomes.^30,32^ Hence, increasing interest in intracellular nucleic acid delivery for improved pro-angiogenic outcomes has been reported,^33,34^ including the delivery of pro-angiogenic genes such as plasmid encoding VEGF (*p*VEGF).^35,36^ Compared to growth factors, gene delivery offers greater stability, reduced off-target side effects and cytotoxicity, lower production costs,^37^ and the ease of encoding a wide variety of genes.^38^ Despite the multiple benefits, the major challenges of gene delivery enclose the need of protecting nucleic acids from degradation while enhancing cellular uptake.^39^ In light of this, we have reinforced *p*VEGF delivery through its combination with the non-viral glycosaminoglycan (GAG)-binding enhanced transduction (GET) peptide system that enhances cellular uptake by integrating cell penetrating and heparan sulfate GAG-binding peptides.^40,41^ Crucially, GET-based formulations have been engineered to result in higher transfection efficiency *in vitro* and *in vivo.*^34,42–45^ Furthermore, we have focused on coupling these GET nano-formulations and biomaterial scaffolds for the efficient and safe delivery of nucleic acids to address tissue-specific challenges. These gene-activated scaffolds have been established as platforms capable of safe, efficient and localized gene delivery with enhanced therapeutic outcomes,^34–36,46–50^ effectively providing tailored biomaterials to enhance pro-regenerative environments in chronic wounds. Here, we have developed a VEGF-activated scaffold with the potential to provide a multifaceted response that enhances both angiogenesis and nerve repair in wound healing. Specifically, the individual aims of this study focused on the formulation and physicochemical characterization of a gene delivery system, comprising *p*VEGF and the non-viral GET peptide, denoted as G-VEGF. This was followed by the therapeutic evaluation of VEGF expression and cell viability of the VEGF complexed GET scaffold-mediated delivery, and compared against the commercially available lipofectamine 3000 delivery vector, resulting in reduced cytotoxicity and enhanced therapeutic outcomes in dermal cells. Finally, the pro-angiogenic and pro-neurogenic potential of the VEGF-activated scaffolds was assessed in established pro-angiogenic models through the cross-talk of dermal fibroblasts and endothelial cells, and *in vitro* growing neurons and *ex vivo* axonal injury neurogenic models.

## 2. Experimental section

All reagents were purchased from Sigma Aldrich (Ireland) unless otherwise stated. All cell culture was performed at 37 °C and 5% CO_2_ unless otherwise stated.

### 2.1. *p*DNA nanoparticles characterization

#### Plasmid propagation

Plasmids encoding vascular endothelial growth factor (*p*VEGF) and green fluorescent protein (*p*GFP) were purchased from Genecopoeia (USA), all under the control of the cytomegalovirus (CMV) promoter. Plasmids were propagated by transforming One Shot TOP10 Chemically Competent *E. coli* bacterial cells according to the manufacturer's protocol and then purified and collected using the Endotoxin free Maxi-prep kit (Qiagen, UK).

#### *p*DNA nanoparticle formulation

G-VEGF nanoparticles were formulated through the electrostatic interaction of the positively charged GET peptide and the negatively charged *p*DNA with a fixed charge ratio of 6 : 1 (CR6) following a previously established method.^43^ Briefly, GET, *p*GFP or *p*VEGF were diluted in OptiMEM™ I Reduced Serum Medium (Biosciences Ireland) and nanoparticles were formulated by the combination of GET and *p*GFP (G-GFP) or *p*VEGF (G-VEGF) for 30 min before use.

#### Physicochemical characterization of VEGF nanoparticles

Physicochemical characterization of the G-VEGF nanoparticles was carried out by dynamic light scattering (DLS) (Zetasizer 3000 HS, Malvern, UK) and nanoparticle tracking analysis (NTA) (NanoSight NS300, Malvern, UK) to determine zeta potential and size, respectively. For the analysis of zeta potential, G-VEGF nanoparticles were prepared with molecular grade water (MG-H_2_O), the volume was increased to 1 mL and transferred to a disposable folded capillary cell (Malvern, UK) before analysis. For NTA size measurements, G-VEGF nanoparticles were prepared as for zeta potential analysis. Data was captured with a sCMOS camera and a Blue488 laser. Data evaluation was carried with the NTA 2.3 software (Malvern, UK).

#### Stability of VEGF nanoparticles in physiological conditions

Stability of the G-VEGF nanoparticles when exposed to physiological conditions was visualized through gel electrophoresis in a 1% agarose gel. Initially, G-VEGF nanoparticles were formulated with molecular grade water (MG H_2_O), divided into 1 μg *p*DNA aliquots and stored at 4 °C or incubated at 37 °C for 1 h with 8 units of DNase I per 1 μg of DNA or 10% fetal bovine serum (FBS). All samples were then loaded on the gel and run for 1 h at 80 V. Samples were loaded as follows from left to right: 1 – Ladder; 2 – *p*VEGF alone; 3 – *p*VEGF alone in serum; 4 – *p*VEGF alone in DNase; 5 – G-VEGF; 6 – G-VEGF in serum; 7 – G-VEGF in DNase. Gels were imaged using an Amersham Imager 680 blot (GE Healthcare).

### 2.2. Cell isolation and culture

Normal human dermal fibroblasts (HDFs) from adult donor were purchased from PromoCell (Germany) and cultured in growth media containing low glucose (1.0 g L^−1^) Dulbecco's Modified Eagles Medium (DMEM) supplemented with 10% FBS and 1% penicillin–streptomycin (P/S). Cells were used between P7–P10 for all experiments.

Human umbilical vein endothelial cells (HUVECs) were purchased from Lonza (Switzerland) and cultured in endothelial growth medium-2 (EGM-2) supplemented with SupplementMix (PromoCell, Germany). Cells were used between P4–P6 for all experiments.

NSC34 murine derived motor-neuron cells (NSC34) were purchased from American type culture collection (ATCC, USA). Cells were cultured in growth medium consisting of DMEM supplemented with 10% FBS, 1% l-glutamine, and 1% P/S, until confluent. Differentiation medium for NSC34 cells consisted of 1 : 1 DMEM Ham's F12, 1% FBS, 1% modified Eagle's non-essential amino acids (Gibco, UK), 0.5% P/S and 10 × 10^−3^ M ATRA.

To assess the ability of the scaffolds to support axonal growth in a suitable *ex vivo* model of axon injury, dorsal root ganglia (DRGs) from 5-month-old adult female C57BL6 mice (*N* = 9, 385–500 g) were seeded onto the scaffolds. All animals were kindly donated by fellow researchers at the Tissue Engineering Research Group in keeping with the 3Rs and the post-mortem harvesting was carried out under HPRA individual license (AE19127/I259) and with ethical approval from the RCSI research Ethics Committee (REC202005013). DRGs were isolated and dissected using a previously established method.^51^ Approximately, 20–24 DRGs with associated roots were dissected from each animal. Each root was trimmed of its associated nerves using fine micro-scissors (F.S.T. Cat # 15000-08) and carefully seeded on scaffolds before being gently flooded with neurobasal medium with 1% P/S, 1% glutamax and 2% B27-supplement (DRG culture medium) and allowed to grow for 14 days with regular media changes (every 2–3 days).

### 2.3. Assessment of VEGF nanoparticles efficacy in 2D cell monolayers

#### Assessment of dermal fibroblast transfection efficiency with GFP nanoparticles

HDFs were seeded on 12-well culture plates at a density of 5.0 × 10^4^ cells per well 24 h prior to transfection. HDF growth medium was removed from each well before adding the G-GFP nanoparticles at different doses (1–10 μg). Subsequently, an additional 500 μL of growth medium was added to each well and left to incubate for 24 hours to allow for cell transfection.

HDFs were fixed on day 3 post-transfection with 4% paraformaldehyde (PFA) for 15 min at room temperature before staining samples with Hoechst 33342 trihydrochloride, trihydrate (1 : 10 000, Biosciences, Ireland) to identify cell nuclei. Fluorescent microscopy images of the GFP-expressing cells and cell nuclei were taken using a Leica DMIL microscope (Leica Microsystems, Switzerland). Images were analyzed using FIJI software^52^ to calculate transfection efficiency counting nuclei and GFP-expressing cells using the analyze particles function of defined thresholded areas. Transfection efficiency was calculated by dividing the number of GFP-expressing cells by total nuclei number per field of view (FOV).

#### Assessment of cell viability and morphology post-transfection in 2D

To understand the post-transfection effect of G-VEGF nanoparticles on HDF viability and morphology, cell viability assays and microscopy analysis were carried out. Initially, HDFs were seeded on 12-well culture plates at a density of 5.0 × 10^4^ cells per well 24 h prior to transfection. Media was removed and G-VEGF nanoparticles were added (1 μg per well) before 500 μL growth medium to each well and left to incubate for 24 h to allow for cell transfection. In the case of L-VEGF, nanoparticles were added followed by 500 μL of OptiMEM™ for 4 h to allow for cell transfection before replacing with fresh growth medium.

Cell metabolic activity was determined through an Alamar Blue™ Cell Viability assay (Biosciences, Ireland) according to the manufacturer's protocol. Briefly, media was removed and growth media containing 10% Alamar Blue™ reagent was added to the cells (500 μL). Samples were then incubated for 1 h. The supernatant was collected and the fluorescence of each sample was measured in triplicate (ex: 570 nm, em: 585 nm) using an Infinite® 200 PRO plate reader (Tecan Group Ltd, Switzerland). Fluorescence measurements of the transfected groups were calculated in relation to the un-transfected control.

DNA content was measured with a Quant-iT™ PicoGreen™ dsDNA Assay Kit (Biosciences, Ireland). Media was removed and wells were flooded with 1 mL buffer (0.2 M sodium carbonate + 0.1% Triton X-100 in DI H_2_O) to lyse the cells. Samples were then subjected to 3 freeze-thawing cycles at −80 °C before measurements were carried out. Fluorescence measurements (ex: 480 nm, em: 520 nm) were performed using an Infinite® 200 PRO plate reader. Finally, DNA concentration was extrapolated from the standard curve.

Cell viability was visualized with the Live/Dead™ Cell Imaging Kit (Cat # R37601, Biosciences, Ireland). Media was removed and washed with sterile DPBS before adding 500 μL of 1× Live/Dead™ solution. Wells were incubated for 15 min at room temperature and washed 3 times. Fluorescent images were taken using a Leica DMIL microscope. Images were analyzed using FIJI software to calculate cell area coverage. Live (Green) area was measured using the analyze particles function of defined thresholded areas. Cell area coverage was calculated by dividing the measured live area over the total area of the FOV.

#### Enzyme-linked immunosorbent assay (ELISA) for VEGF, bFGF and TGF-β1 quantification post-transfection

Human vascular endothelial growth factor (VEGF), human basic-fibroblast growth factor (bFGF) and human transforming growth factor-β1 (TGF-β1) ELISA kits (R&D Systems, USA) were used to quantify the protein release from HDFs and HUVECs following transfection. ELISAs were carried out as previously reported^51^ with the conditioned media collected on days 1, 3 and 7 post-transfection. Absorbance measurements at 450 and 540 nm were taken using an Infinite® 200 PRO plate reader. Finally, VEGF, bFGF and TGF-β1 expression was calculated by extrapolation from the standard curve.

### 2.4. Assessment of therapeutic response of VEGF-activated scaffolds

#### Collagen-GAG (CG) scaffold fabrication and crosslinking

The CG slurry used for scaffold fabrication was prepared as previously described.^53^ Briefly, a solution combining 0.5% w/v of microfibrillar type I collagen isolated from bovine tendon (Integra Life Sciences, USA) and 0.05% w/v chondroitin-6-sulfate (GAG) isolated from shark cartilage (Sigma-Aldrich, Germany) was prepared using acetic acid (0.05 M) as a solvent. The solution was blended using an Ultra-Turrax® T25 homogenizer (IKA, Germany) at 15 000 rpm at 4 °C. Then, the slurry was degassed under vacuum (∼5 Torr) at room temperature before being stored at 4 °C until use. CG scaffolds were prepared by lyophilization process. Initially, 400 μL slurry were pipetted into 10 mm diameter aluminium molds before freezing to a final temperature of −10 °C, reduced at a rate of 1 °C min^−1^ and maintained for 60 min. Then, the solvent was sublimated under vacuum (200 mTorr) for 24 h at 0 °C.

To enhance their structural properties, CG scaffolds were chemically crosslinked with 1-ethyl-3-(3-dimethyl aminopropyl)-carbodiimide (EDAC) and *N*-hydroxy succinimide (NHS) (Sigma-Aldrich, Germany) as previously described.^53^ Briefly, scaffolds were hydrated in DPBS for 1 h prior to crosslinking. Deionized H_2_O solutions containing EDAC (6 mmol per gram of collagen to be crosslinked) and NHS (2.5 M ratio of EDAC : NHS) were prepared and mixed. Scaffolds were then transferred to the EDAC/NHS solutions and allowed to crosslink for 2 h at room temperature. After crosslinking, scaffolds were washed 3 times with DPBS to remove excess EDAC/NHS before sterilization in 70% ethanol. Finally, scaffolds were washed 3 more times with DPBS under sterile conditions.

#### VEGF-activated scaffold fabrication and cell seeding

CG scaffolds were gene-activated through the soak-loading of *p*VEGF/GET (G-VEGF) or *p*VEGF/lipofectamine (L-VEGF) nanoparticles, using the L-VEGF treatment as a positive control with a ‘gold standard’ commercially available vector. For the gene-activation of G-VEGF scaffolds, G-VEGF nanoparticles were soak-loaded on one side of the CG scaffolds (1 μg *p*VEGF) and incubated for 45 min at 37 °C. Then, scaffolds were flipped and the process was repeated. For the gene-activation of L-VEGF scaffolds, L-VEGF nanoparticles were initially formulated according to the manufacturer's protocol, and soak-loaded as described for G-VEGF.

After soak-loading of G-VEGF or L-VEGF nanoparticles, 1.25 × 10^5^ cells were seeded onto the first side of the scaffolds at a concentration of 2.5 × 10^6^ cells per mL. Scaffolds were allowed to incubate at 37 °C for 15 min before repeating the process on the opposite side. Finally, wells containing G-VEGF scaffolds were flooded with growth media and incubated (37 °C, 5% CO_2_) for 24 h to allow for cell transfection before changing the media in the wells. Wells with L-VEGF scaffolds were flooded with OptiMEM™ for 4 h to allow for transfection before replacing the supernatant with growth medium. CG scaffolds were used as a negative control.

#### Assessment of release behavior from VEGF-activated scaffolds

To assess the *p*VEGF release profile from the VEGF-activated scaffolds, scaffolds were placed on 24-well plates and soak-loaded with 10 μg of *p*VEGF (30 μL) as previously described. Scaffolds were then transferred to a new well-plate and flooded with 2 mL of MG H_2_O. The release profile was determined at 37 °C under static conditions by collecting 200 μL of supernatant at different time points, followed by the addition of 200 μL of fresh MG H_2_O. Then, the supernatant collected was incubated with 50 μL of heparin (1 mg mL^−1^) at 37 °C for 90 min in order to disassociate the nanoparticles. Finally, the *p*VEGF concentration was determined with a Quant-iT™ PicoGreen™ dsDNA Assay Kit.

#### Assessment of cell viability and morphology from VEGF-activated scaffolds

Metabolic activity, DNA content and ELISA measurements of VEGF, bFGF and TGF-β1 growth factors were determined as previously described for 2D experiments, except for Alamar Blue™ reagent volume used (1 mL) and incubation time (2 h). Cell morphology and distribution of HDFs and HUVECs in VEGF-activated scaffolds was analyzed through cytoskeleton (F-actin) and cell nuclei visualization. HDF-seeded scaffolds were fixed in 4% PFA for 1 h at 4 °C before being washed 3 times with DPBS and stored at 4 °C. Scaffolds were permeabilized with 0.1% Triton X-100 solution for 5 min followed by incubation at room temperature with Alexa Fluor 555 Phalloidin™ (1 : 500) for 2 h and Hoechst 33342 (1 : 10 000) for 15 min with 3 DPBS washes in between steps. HUVEC-seeded scaffolds were fixed and permeabilized as before followed by 2 h incubation in 1% bovine serum albumin (BSA). Scaffolds were then incubated with mouse anti-CD31 (1 : 100, Cat # sc-376764 AF 488, Santa Cruz Biotechnologies, USA) overnight at 4 °C. Finally, scaffolds were washed with DPBS, stained with Alexa Fluor 555 Phalloidin™ (1 : 500) and Hoechst 33342 (1 : 10 000) as before. All scaffolds were imaged using a Zeiss LSM 710 confocal microscope, maintaining consistent gain, exposure, and magnification.

### 2.5. Assessment of pro-angiogenic potential of VEGF-activated scaffolds

To understand the ability of the VEGF-activated scaffolds to direct the migration and organization of endothelial cells through paracrine signaling, scratch and tube formation assays were performed utilizing the conditioned media derived from HDF-seeded scaffolds. Endothelial cell migration was assessed through a scratch assay. Briefly, 5.0 × 10^4^ HUVECs were seeded on 12-well plates 48 h prior to use. Media was removed and a scratch was manually performed with a P1000 pipette tip. All wells were washed and conditioned media from CG, G-VEGF and L-VEGF scaffolds were added to wells (1 mL). Controls including no VEGF supplementation (VEGF−), 10% FBS supplementation (FBS+) and VEGF supplementation (VEGF+) were also used before transferring the plate to a Zeiss Celldiscoverer 7 microscope. Images were taken every hour for 48 h. Cell migration was calculated as the change in area from the scratch at different timepoints in relation to the area at the start timepoint (0 h).

Having assessed the differences in cell migration, a tube formation assay was carried out to analyze the effect of the VEGF-activated scaffolds to influence the ability of HUVECs to form vascular-like structures. Geltrex™ LDEV-Free Reduced Growth Factor Basement Membrane Matrix (Biosciences, Ireland) was thawed 24 h prior to use at 4 °C. Following this, a 96-well plate was coated with 80 μL of Geltrex. The plate was then centrifuged and incubated at 37 °C for 30 min to gel. Next, 6.0 × 10^4^ HUVECs were resuspended for each group in 600 μL of conditioned media and controls. After incubation, 200 μL of conditioned media cell-suspensions were added to the Geltrex-coated wells in triplicate. The plate was transferred to a Zeiss Celldiscoverer 7 microscope and images were taken every hour for 48 h. Image analysis was carried out using the Angiogenesis Analyzer for ImageJ^54^ to determine the total branch points, tube number, tube length and isolated segments formed in each group.

### 2.6. Assessment of VEGF-activated scaffolds in a HDF/HUVEC co-culture model without exogenous growth factor supplementation

A co-culture model with HDFs and HUVECs was used to analyze the effect of VEGF-activated scaffolds on cell organization and viability as a model of the environment that cells experience in chronic wounds where no exogenous VEGF is supplemented. Briefly, VEGF-activated scaffolds were prepared and seeded with 1.25 × 10^5^ cells (1 : 2, HDF : HUVEC) on each side at a concentration of 2.5 × 10^6^ cells per mL. Wells were then flooded with 1 mL co-culture media (1 : 1, DMEM + 10%FBS + 1% P/S : EBM-2) and cultured for up to 14 days. Metabolic activity and DNA content were assessed as previously described. Cell morphology was visualized using Alexa Fluor 555 Phalloidin™ (1 : 500), Hoechst 33342 (1 : 10 000) and mouse anti-CD31 (1 : 100) to distinguish between endothelial cells (CD31+) and dermal fibroblasts (CD31−).

### 2.7. Characterization of pro-neurogenic ability of VEGF-activated scaffolds through *in vitro* and *ex vivo* axonal injury model

To assess the neurogenic ability of the VEGF-activated scaffolds, *in vitro* young growing neurons (NSC34) and *ex vivo* injured adult axons in dorsal root ganglia (DRG) models were used. Initially, VEGF-activated scaffolds were prepared before seeding each side with 1.25 × 10^5^ NSC34 cells at a concentration of 2.5 × 10^6^ cells per mL. Scaffolds were cultured in growth medium for 3 days before switching to differentiation medium for the following 7 days. Metabolic activity was measured after 1, 3, 7 and 10 days before collecting the scaffolds on day 10 for DNA content analysis. Different NSC34-seeded scaffolds were fixed with 4% PFA for 1 h at room temperature, washed with DPBS and stored at 4 °C. These scaffolds were then permeabilized with 0.1% Triton X-100 for 30 min before overnight incubation with β-tubulin III antibody (1 : 500), Atto-Phalloidin (1 : 500) for 2 h, and 4′,6-diamidino-2-phenylindole (DAPI) (1 : 1000) for 1 h; all at room temperature. Finally, representative images of each scaffold were taken using a Zeiss LSM 710 confocal microscope for quantitative analysis. Images were analyzed using FIJI software to create max intensity *z*-projections. The neurogenic capacity of the scaffolds to promote neurite outgrowth was assessed by measuring the max neurite length per FOV using a manual tracing tool. β-Tubulin III intensity and scaffold area coverage were measured using the analyze particles function of defined thresholded areas.

To assess the capacity of the scaffolds to promote the growth of injured adult axons, an *ex vivo* DRG explant model was used. Briefly, a single DRG was placed on the middle of the scaffolds. Each scaffold was then immersed in 400 μL of DRG culture medium and incubated for 12 h to allow the DRGs to adhere. Following this, the volume of media was increased to 750 μL and half was replaced every second day. After 14 days of culture, metabolic activity was analyzed before scaffolds were washed twice with DPBS and fixed in 4% PFA for 1 h at room temperature. Scaffolds were then washed and stained using rabbit anti-β-tubulin III antisera (1 : 500) and DAPI (1 : 1000). Scaffolds were imaged using a Zeiss Examiner.Z1 confocal microscope. Analysis was performed similarly as described for NSC34 for calculating the max neurite length. Average neurite length was analyzed by calculating the straight-line distance from axonal growth cone to the DRG body.

### 2.8. Statistical analysis

Statistical analysis was carried out using Graph-Pad Prism v 10.0.2. One-way ANOVA with a Tukey *post-hoc* test was used when more than one treatment was compared. Two-way ANOVA with Bonferroni *post-hoc* test was used when more than one treatment was compared across two factors. All experiments were performed in triplicate and results are expressed as mean ± standard deviation (SD).

## 3. Results

### 3.1. VEGF nanoparticles presented suitable physicochemical properties and stability for cellular internalization

**Fig. 1:**
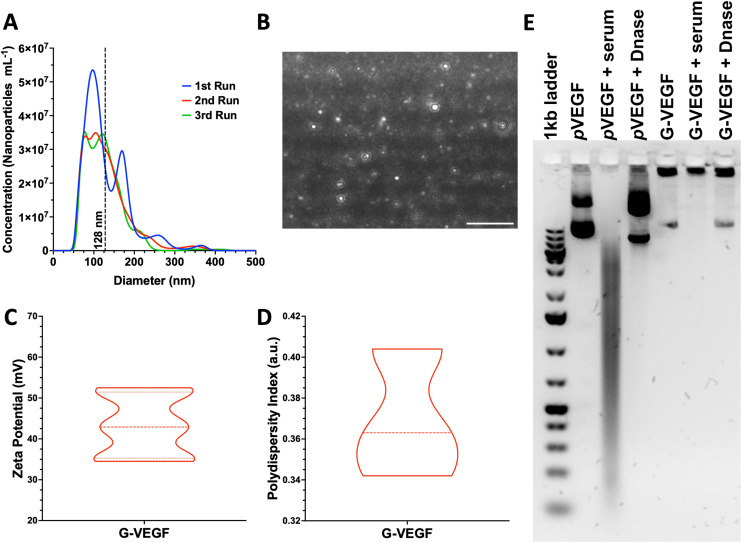
G-VEGF nanoparticles present optimal physicochemical properties while protecting *p*VEGF from physiological degradation. (A) Size distribution of G-VEGF nanoparticles determined through laser tracking analysis. (B) Representative image of G-VEGF nanoparticles in suspension when analyzed through laser tracking software. (C and D) Characterization of G-VEFG nanoparticles charge and polydispersity index showing the distribution of the properties through dynamic light scattering. (E) Agarose gel electrophoresis characterization of G-VEGF nanoparticles and naked *p*VEGF when exposed to physiological-like conditions, including serum and DNase interactions. Data shows mean ± SD (*n* = 6). Scale bar in B = 2 μm.

Size and charge of nanoparticles are essential properties that influence cellular internalization.^55^ Thus, analysis of these physicochemical properties of *p*VEGF/GET (G-VEGF) nanoparticles was carried out, revealing a suitable diameter and charge for cellular transfection. G-VEGF nanoparticles exhibited a diameter of 128.8 ± 54.1 nm (Fig. 1A), charge of 43.3 ± 7.3 mV (Fig. 1C), and polydispersity index of 0.36 ± 0.03 (Fig. 1D), with a rounded morphology as visualized through laser tracking analysis (Fig. 1B). Moreover, the analysis of G-VEGF transfection efficiency on HDFs displayed a dose-dependent increase in efficiency (ESI Fig. 1†)

Having determined the optimal physicochemical properties of the G-VEGF nanoparticles, we investigated their stability in physiological conditions (Fig. 1E). The stability of the G-VEGF nanoparticles remained unchanged (Fig. 1E, lane 5), even when subjected to serum and DNase at 37 °C for 1 h (Fig. 1E, lane 6 and 7, respectively), indicating the successful shielding of the *p*VEGF by the GET peptide under physiological conditions. In contrast, naked *p*VEGF exposed to the same conditions, exhibited a clear drift of the negatively charged plasmid towards the positively charged cathode when analyzed alone, in serum, and DNase, revealing the vulnerability of the naked *p*DNA when not complexed with GET (Fig. 1E, lane 2, 3 and 4, respectively).

### 3.2. Transfection of dermal fibroblasts with VEGF nanoparticles resulted in enhanced VEGF expression

**Fig. 2:**
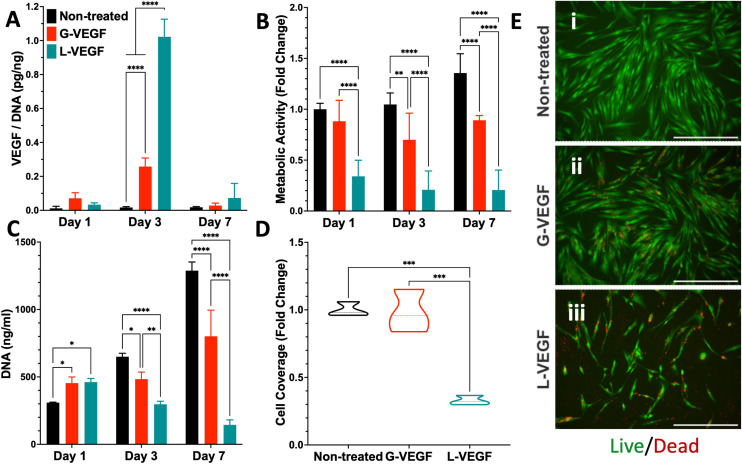
Treatment of HDFs with G-VEGF nanoparticles enhances VEGF expression without substantially compromising cell viability in an *in vitro* monolayer. (A) Quantification of VEGF expression per unit DNA in the supernatant collected from HDFs after VEGF nanoparticle treatments. (B) Assessment of metabolic activity and (C) DNA quantification from HDFs post-transfection. (D) Quantitative analysis of cell coverage of live cells after Live/Dead staining on transfected HDFs. (E) Representative images of Live/Dead staining on non-treated, G-VEGF-treated, and L-VEGF-treated HDFs. Data shows mean ± SD (*n* = 6) and * indicates *p* < 0.05, **p < 0.01, ***p < 0.001, ****p < 0.0001. Scale Bars in E = 500 μm.

Following characterization of the G-VEGF nanoparticles, we investigated the biological outcome of G-VEGF delivery (1 μg *p*VEGF) to *in vitro* monolayers of HDFs. G-VEGF delivery led to an approximate 10-fold increase on VEGF expression compared to the non-treated control (Fig. 2A). However, L-VEGF delivery (1 μg *p*VEGF) resulted in a ∼40-fold increase compared to the non-treated control and a ∼4-fold increase compared to the G-VEGF treatment.

Subsequent evaluation of cytotoxicity of the non-treated, G-VEGF and L-VEGF-treated HDFs was performed over 7 days and revealed important differences. Analysis of metabolic activity demonstrated a slight reduction in cell viability with the G-VEGF treatment while the metabolic activity of the L-VEGF-treated HDFs exhibited a pronounced cytotoxic response on days 1, 3 and 7 post-transfection compared to the non-treated HDFs (Fig. 2B). Non-treated and G-VEGF-treated cells exhibited a continuous increase in cell proliferation over 7 days as measured by DNA quantification, although proliferation of G-VEGF-treated HDFs was comparatively lower. Conversely, cell proliferation with the L-VEGF nanoparticles resulted in a significant reduction over 7 days compared to both non-treated and G-VEGF-treated HDFs.

Further analysis of cell viability involved the visualization and quantification of dermal fibroblast viability with the Live/Dead assay. Relative to non-treated cells, significantly enhanced cell coverage was observed following G-VEGF treatment (98.2 ± 15.8%) compared to the L-VEGF treatment (32.8 ± 3.4%) (Fig. 2D). The Live/Dead images also aligned with the analysis of metabolic activity and DNA content, showing a greater number of live (green) cells in the non-treated and G-VEGF groups compared to the L-VEGF treatment (Fig. 2E).

### 3.3. VEGF-activated scaffolds enhanced VEGF expression in dermal fibroblasts and improved endothelial cell organization

**Fig. 3:**
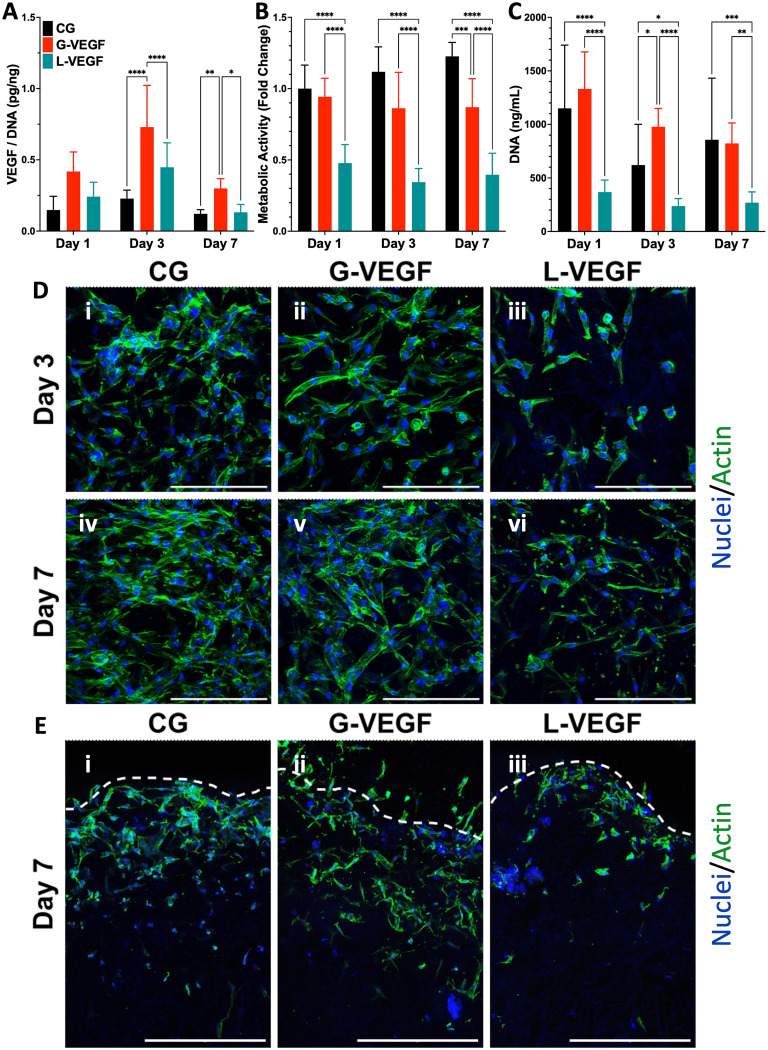
VEGF-activated scaffolds enhance VEGF expression in dermal fibroblasts without compromising cell viability up to 7 days. (A) Quantification of VEGF expression per unit DNA in the supernatant collected from the HDF-seeded gene-free and VEGF-activated scaffolds. (B) Metabolic activity and (C) DNA content quantification of HDF-seeded scaffolds. (D) Representative surface confocal images of HDF-seeded scaffolds showing differences in cellular morphology and organization after days 3 and 7 post-transfection. (E) Representative cross-section confocal images of sliced hDF-seeded scaffolds on day 7 post-transfection – Segmented line represents the superior edge of the scaffold. Data shows mean ± SD (*n* = 9) and * indicates *p* < 0.05, **p < 0.01, ***p < 0.001, ****p < 0.0001. Scale bars in D = 200 μm and E = 500 μm.

Having established the potential of G-VEGF nanoparticles as a therapeutic capable of enhancing pro-angiogenic growth factor expression without compromising cell viability in 2D HDF monolayers, we next evaluated the biological effect of G-VEGF nanoparticles when incorporated into the CG scaffold (G-VEGF scaffolds). Initially, characterization of the dsDNA release behavior from the G-VEGF scaffolds showed that most of the cargo is retained within the scaffolds and only ∼4% of the loaded dsDNA is released (ESI Fig. 3†) which falls in accordance to the behavior of similar platforms previously reported from our lab.^46^ We then assessed the influence of the scaffolds on the expression of critical pro-angiogenic growth factors (VEGF, basic fibroblast growth factor (bFGF) and transforming growth factor-β1 (TGF-β1)) from HDFs and HUVECs over 7 days (Fig. 3A and ESI Fig. 4†). Crucially, G-VEGF scaffolds induced a ∼3.5-fold increase in VEGF expression compared to CG scaffolds on day 3. However, the expression of bFGF (8-fold) and TGF-β1 (10-fold) was significantly higher in L-VEGF scaffolds, despite reduced VEGF expression at the same time point.

Importantly, the increased VEGF expression elicited by G-VEGF scaffolds did not compromise HDF viability, unlike L-VEGF scaffolds. Assessment of metabolic activity revealed a substantial decrease in cell viability when HDFs were cultured in L-VEGF scaffolds (47.8 ± 12.9, 34.4 ± 9.6 and 39.6 ± 15.1% on days 1, 3 and 7, respectively) aligning with the outcomes observed in 2D monolayers. In contrast, trends in metabolic activity observed with G-VEGF scaffolds closely mirrored those of the CG only scaffold, indicating no cytotoxic response with this treatment (Fig. 3B). Additionally, a similar trend in HDF proliferation was observed within G-VEGF scaffolds compared to CG scaffolds on days 1, 3, and 7 (Fig. 3C). By day 7, a ∼3-fold increase in proliferation was observed within G-VEGF scaffolds compared to L-VEGF scaffolds.

The impact of VEGF-activated scaffolds on HDF growth was further assessed by confocal microscopy. On day 3 post-transfection, HDFs exhibited improved distribution and maintained an elongated spindle-like morphology, indicative of a healthy pro-regenerative phenotype,^56^ on CG and G-VEGF scaffolds as demonstrated by F-actin staining (Fig. 3D, i and ii) compared to HDFs on L-VEGF scaffolds (Fig. 3D, iii). By day 7 post-transfection, a greater number of cells were evident on these scaffolds, indicating increased proliferation while maintaining the spindle-like morphology and distribution observed on day 3 (Fig. 3D, iv and v). In contrast, cell number and distribution of HDFs on L-VEGF scaffolds were notably lower on day 3 as demonstrated by nuclei staining, indicating a lack of cell proliferation and evidence of toxicity on day 7 (Fig. 3D, iii and vi). Visualization of scaffold cross-sections by confocal microscopy demonstrated that both HDFs distribution and infiltration were greater in the CG and G-VEGF scaffolds compared to the L-VEGF (Fig. 3E), indicating the unaltered migratory activity of HDFs correlated to healthy native tissue.

Having confirmed that G-VEGF scaffolds significantly enhanced VEGF expression in HDFs and displayed reduced cytotoxicity compared to L-VEGF scaffolds, we proceeded to evaluate the response from HUVECs when seeded on the G-VEGF scaffolds. The expression of pro-angiogenic growth factors from HUVEC-cultured scaffolds was assessed over a 7-day period. Measurement of VEGF, bFGF and TGF-β1 expression revealed an amplified effect on bFGF and TGF-β1 production with G-VEGF scaffolds compared to both CG only and L-VEGF scaffolds. However, VEGF expression was significantly enhanced (∼25-fold) with L-VEGF scaffolds over CG and G-VEGF scaffolds (ESI Fig. 5†).

**Fig. 4:**
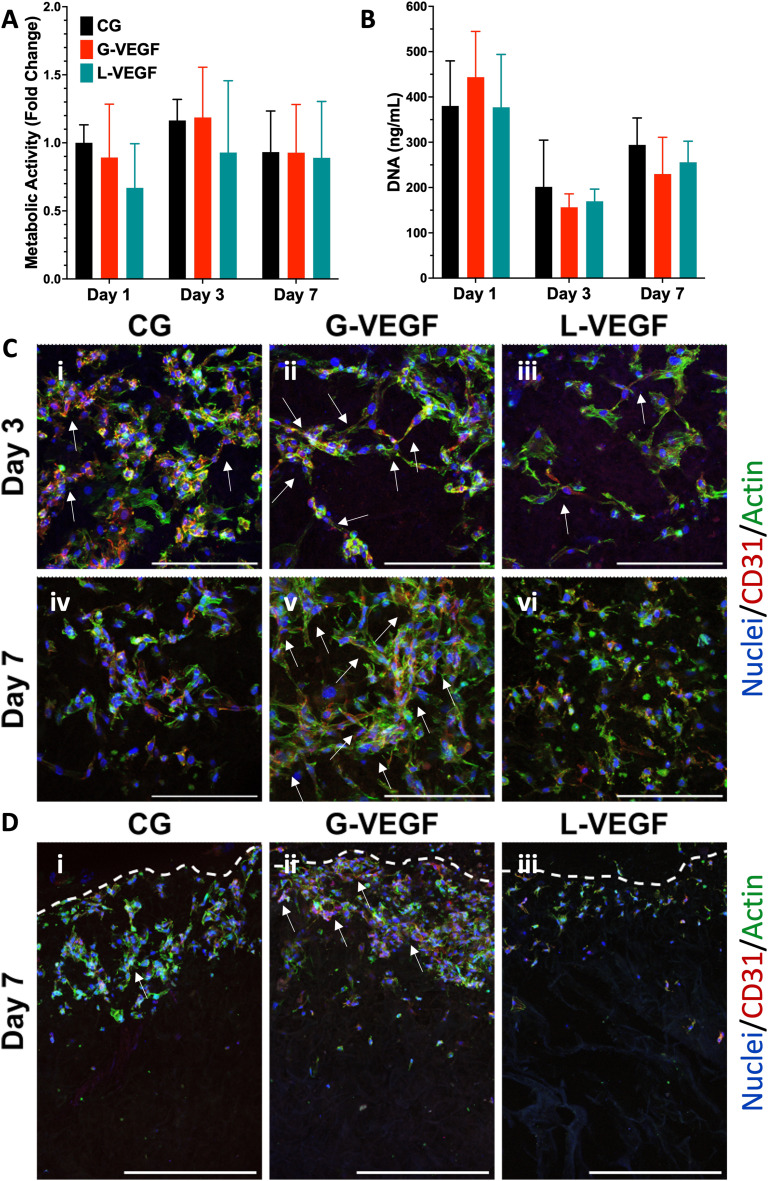
VEGF scaffolds facilitate endothelial cell organization without affecting cell viability. (A) Normalized metabolic activity and (B) DNA content of HUVEC-seeded gene-free and VEGF-activated scaffolds. (C) Representative surface confocal images of HUVEC-seeded scaffolds highlighting cellular organization after days 3 and 7 post-transfection. (D) Representative cross-section confocal images of sliced HUVEC-seeded scaffolds on day 7 post-transfection, highlighting cellular infiltration – Segmented line represents the edge of the scaffold. Data shows mean ± SD (*n* = 6). Scale bars in C = 200 μm and D = 500 μm.

Subsequent characterization of cell viability by measurement of metabolic activity of HUVECs cultured on VEGF-activated scaffolds did not display statistical differences between groups, unlike the observations made with the HDFs-seeded scaffolds. Assessment of metabolic activity over a 7-day period did not reveal any statistically significant disparities amongst CG, CG-G-VEGF and L-VEGF scaffolds (Fig. 4A). Moreover, these results were corroborated by DNA quantification, demonstrating similar trends among the three groups analyzed over the 7-day period (Fig. 4B). However, comprehensive evaluation of confocal images from HUVECs in scaffolds revealed discernible differences in cell morphology, distribution, and organization amongst CG, G-VEGF and L-VEGF scaffolds. On day 3 post-transfection, both CG and G-VEGF scaffolds exhibited a higher number of endothelial cells per field of view compared to L-VEGF scaffolds (Fig. 4C, i–iii). Notably, organization of HUVECs into vascular-like structures was considerably improved on G-VEGF scaffolds (white arrows). By day 7 post-transfection, signs of improved HUVECs distribution, number and organization were evident on G-VEGF scaffolds, while both CG and L-VEGF scaffolds displayed lower numbers and decreased cell organization (Fig. 4C, iv–vi). Cell infiltration was also visualized through confocal imaging of scaffold cross-sections on day 7 post-transfection (Fig. 4D). As anticipated, an increase in HUVEC infiltration was observed in CG and G-VEGF scaffolds. However, prominent vascular-like structures were observed in the G-VEGF scaffolds.

### 3.4. Pro-angiogenic ability of endothelial cells is enhanced by dermal fibroblasts cultured on VEGF-activated scaffolds

**Fig. 5:**
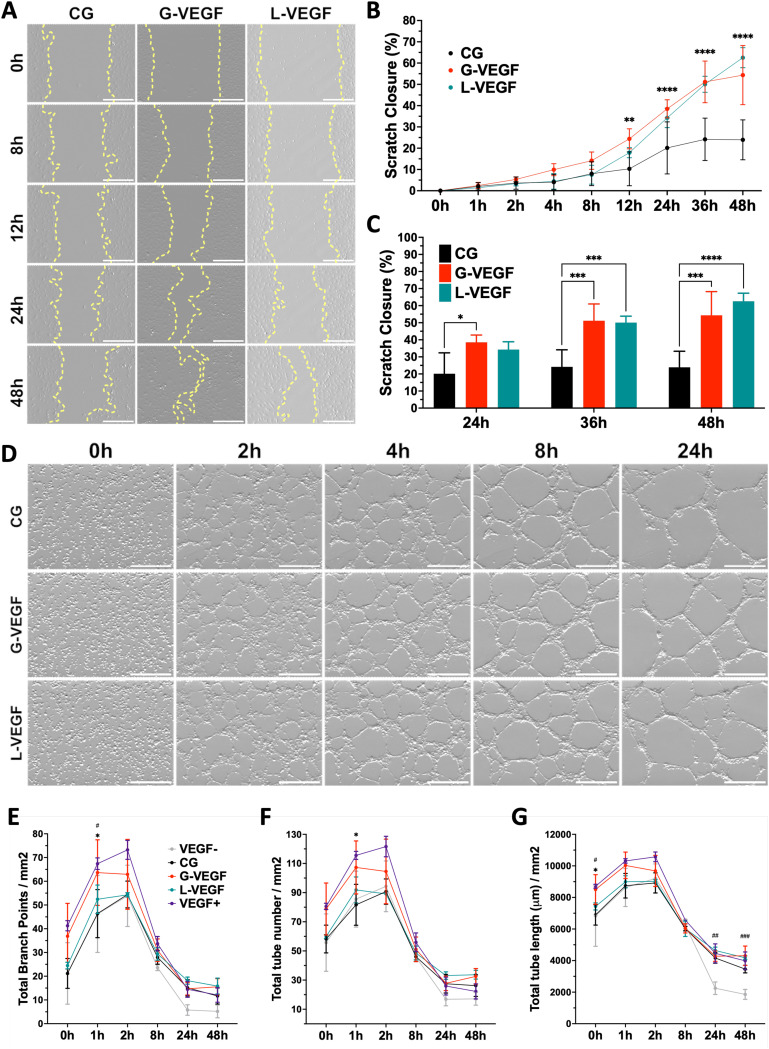
Pro-angiogenic behavior of endothelial cells is enhanced when treated with the supernatant from fibroblast-seeded VEGF-activated scaffolds. (A) Representative bright field images of scratched HUVEC monolayers treated with supernatant from HDF-seeded scaffolds – yellow lines trace cell migration. (B) Quantitative analysis of scratch closure up to 48 hours, and (C) detailed statistical differences between groups on the last 24 hours of cell migration. (D) Representative bright field images of tube formation assay with HUVECs exposed to supernatant from HDF-seeded scaffolds. Quantification of (E) total branch points (F) total tube number and (G) total tube length from vascular-like structure formed through HUVEC organization. Data shows mean ± SD (*n* = 4), * in C indicates *p* < 0.05, ***p < 0.001, ****p < 0.0001, # in E, F and G indicates comparison of G-VEGF *vs.* VEGF−, #*p* < 0.05, ##*p* < 0.01, ###*p* < 0.001, and * in B, E, F and G indicates comparison of G-VEGF *vs.* CG, *p < 0.05. Scale bars in A and D = 500 μm.

Having established the pro-angiogenic potential of G-VEGF scaffolds on HDFs and HUVECs, we next focused on the regenerative effect of growth factors released by HDFs on the migration and tube formation ability of HUVECs. A scratch assay assessing endothelial cell migration revealed significantly accelerated cell migration by HUVECs treated with the supernatant from HDF-cultured G-VEGF scaffolds (Fig. 5A). Quantification of scratch closure demonstrated a ∼2.5-fold increase in cell migration on the G-VEGF group at 12 h compared to the CG group which was maintained up to 48 hours (Fig. 5B). Notably, the supernatant from L-VEGF scaffolds elicited a similar response to the G-VEGF group in terms of scratch closure, showing a significant increase as early as 24 h compared to the CG group (Fig. 5C).

Following the observed effect on cell migration elicited with the supernatant from G-VEGF scaffolds, we assessed its impact on HUVECs tube formation. The supernatant from G-VEGF scaffolds induced increased tube development compared to the CG group (Fig. 5D). Quantification of branch points (Fig. 5E), tube number (Fig. 5F) and tube length (Fig. 5G) revealed a significant increase in branching and tube formation at earlier time points (1 h) and the maintenance of tube length (up to 48 h) compared to the CG and VEGF- (no VEGF supplementation) groups. While the trends observed with the L-VEGF group were similar to the G-VEGF group, the overall average observed was lower.

### 3.5. VEGF-activated scaffolds promote the organization of endothelial cells in a 3D *in vitro* co-culture model without exogenous VEGF supplementation

**Fig. 6:**
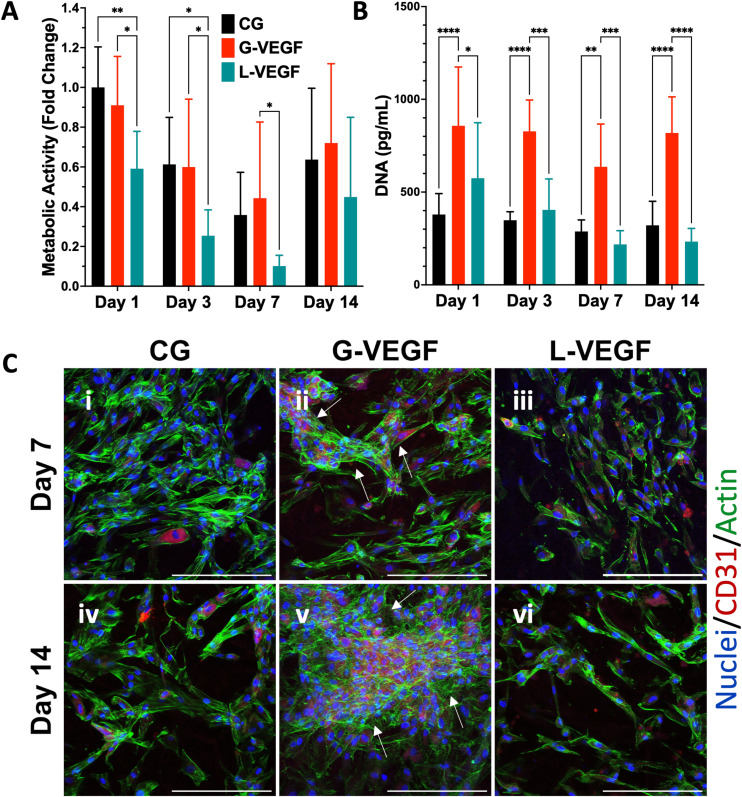
VEGF-activated scaffolds increased vascular network formation by endothelial cells in a co-culture model with dermal fibroblasts. (A) Quantification of metabolic activity and (B) DNA content of co-culture (HDFs and HUVECs)-seeded, gene-free, and VEGF-activated scaffolds. (C) Representative confocal images of scaffolds on days 7 and 14 post-transfection, highlighting endothelial cell organization through CD-31 staining. Data shows mean ± SD (*n* = 6) and * indicates *p* < 0.05, **p < 0.01, ***p < 0.001, ****p < 0.0001. Scale bars in C = 200 μm.

Following the assessment of the indirect effect of G-VEGF scaffolds on the pro-angiogenic functional properties of HUVECs, we evaluated the direct interaction between HDFs and HUVECs in an *in vitro* co-culture model deprived of exogenous pro-angiogenic growth factor supplementation as a more relevant model of the *in vivo* scenario in chronic wounds. Analysis of cell growth, by measuring increases in cell metabolic activity of the co-cultures on scaffolds revealed a consistent trend between CG and G-VEGF scaffolds (Fig. 6A). In contrast, the metabolic activity of the L-VEGF co-culture exhibited a diminished trend compared to the other groups over a 14-day period. Further quantification of DNA revealed a reduced amount of DNA in both CG and L-VEGF groups over the 14-day period compared to G-VEGF scaffolds (Fig. 6B), with DNA measurements reaching their lowest on day 7 for all groups.

To explore further the impact of the VEGF-activated scaffolds on the angiogenic process in a co-culture model, we examined cell morphology and distribution within the scaffolds by confocal microscopy (Fig. 6C). Significantly, a higher number of CD31-expressing cells (HUVECs) were observed on the G-VEGF scaffolds compared to CG and L-VEGF on days 7 (ii) and 14 (v). Moreover, greater cellular organisation in the form of vascular-like structures were evident on day 7 (white arrows), a feature that persisted up to day 14. In contrast, the number, distribution and organisation of CD31-expressing cells was much lesser in both CG and L-VEGF scaffolds, underscoring insufficient angiogenic support from the these scaffolds on both days 7 (i, iii) and 14 (iv, vi).

### 3.6. VEGF-activated scaffolds demonstrate enhanced neurotrophic potential to promote neurite growth in growing neurons and injured adult neural tissue explants

**Fig. 7:**
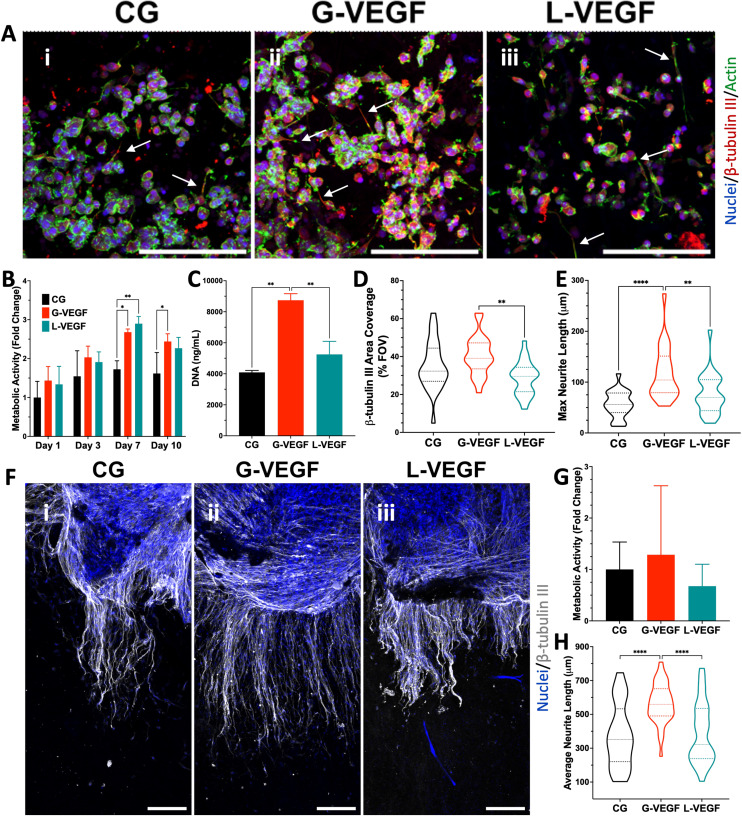
VEGF-activated scaffolds enhanced neurite extension from growing neurons *in vitro* and from dorsal root ganglia explant neurons. (A) Representative confocal images of neurons grown on VEGF-activated scaffolds 10 days after seeding. (B) Quantification of metabolic activity and (C) DNA content of neuron-seeded scaffolds. (D) Quantitative analysis of % area of scaffold coverage and (E) max neurite length of neurons on VEGF-activated scaffolds after 10 days of growth. (F) Representative confocal images of adult mouse dorsal root ganglia (DRG) grown on different scaffold groups for 14 days. (G) Analysis of metabolic activity at day 14 from dorsal root ganglia on scaffolds. (H) Quantification of dorsal root ganglia neurite on gene-free and VEGF-activated scaffolds. Data shows mean ± SD (*n* = 3) and * indicates *p* < 0.05, **p < 0.01, ***p < 0.001, ****p < 0.0001. Scale bars in A and F = 200 μm.

Having established the vascular potential of the VEGF-activated scaffolds, we next investigated the capacity of the scaffolds to promote neurite outgrowth. Fluorescent images of mouse motor neurons-seeded scaffolds revealed that, while all scaffold groups supported neuronal growth, G-VEGF scaffolds exhibited the largest areas of β-tubulin III (an exclusive neuronal microtubule marker) positive cells, with more neurites observed extending across the scaffolds (Fig. 7A). In contrast, neurons cultured in L-VEGF scaffolds displayed lower cell numbers and reduced scaffold area coverage. Evaluation of neuronal metabolic activity demonstrated the ability of G-VEGF scaffolds to significantly enhance long-term cell metabolic activity compared to CG scaffolds at day 10 (Fig. 7B). Moreover, DNA content analysis revealed that G-VEGF scaffolds promoted significant neuronal proliferation compared to both CG and L-VEGF scaffolds (Fig. 7C). Quantification of β-tubulin III area percentage (Fig. 7D) demonstrated that G-VEGF scaffolds facilitated superior coverage compared to L-VEGF scaffolds. Additionally, the neurotrophic capacity of G-VEGF scaffolds was demonstrated and showed significant enhancements in neurite length compared to both CG and L-VEGF scaffolds (Fig. 7E).

## 4. Discussion

The overarching aim of this study involved the development of a VEGF-activated scaffold for the promotion of pro-angiogenic and pro-neurogenic signaling for chronic wound healing. Initially, the physicochemical properties of the VEGF complexed GET nanoparticles were characterized resulting in the formulation of a gene-delivery system with suitable properties and capable of protecting the genetic cargo from physiological degradation. *In vitro*, dermal fibroblasts exposed to the complexed nanoparticles produced higher amounts of VEGF protein without a compromise in cell viability. Encouraged by this pro-angiogenic outcome, dermal fibroblasts and endothelial cells were cultured on the scaffolds resulting in enhanced VEGF expression and improved endothelial cell migration and organization into vascular-like structures. Finally, the neurogenic ability of the scaffolds was assessed using an *in vitro* model of moto-neurons and *ex vivo* adult dorsal root ganglia, revealing increased cell viability, neurite formation and extension in both models. Taken together, our study demonstrates that the VEGF-activated scaffolds can induce pro-angiogenic and pro-neurogenic responses from dermal, vascular and neural cells, showcasing the potential of this platform for the healing of chronic wounds.

In order to develop the platform, the *p*VEGF/GET (G-VEGF) nanoparticles were characterized through physicochemical techniques to ensure the optimal charge, size, polydispersity index and morphology for successful cellular internalization and efficient transfection. Importantly, G-VEGF nanoparticles presented optimal properties for successful internalization as nanoparticles with a diameter smaller than 200 nm and a positive charge (>+10 mV) are likely to undergo clathrin-mediated endocytosis and exhibit susceptibility to endosomal escape.^55,57^ Additionally, the average hydrodynamic size and charge of G-VEGF nanoparticles align with previous literature,^43^ where properties of GET-based nanoparticles were finely tuned for increased cellular delivery.

Following physicochemical assessment of the G-VEGF nanoparticles, their therapeutic influence on pro-angiogenic growth factor expression by fibroblasts was characterized while comparing its response against the commercially available lipofectamine 3000 (L-VEGF) nanoparticles. Notably, treatment of fibroblasts in 2D environments with VEGF complexed GET nanoparticles elicited a higher VEGF expression to cell viability balance compared to lipofectamine delivery. The heparan-binding motif and polyarginine sequence of the GET vector may enhance the endocytic uptake and intracellular transduction of the plasmid,^40^ leading to more pronounced effects of the delivered gene cargo with minimal cytotoxic effects. In contrast, lipofectamine 3000 is a liposome-based vector considered to be the non-viral transfection ‘gold standard’ due to its ability to avoid lysosomal degradation.^58^ However, plasmid delivery with lipofectamine 3000 has shown significant cytotoxicity despite greater transfection efficiency against other commercial vectors in different cell types.^59^ Overall, these findings highlight the importance of vector optimization for effective transfection of therapeutic gene cargo with minimal cytotoxic effects, with GET shown to be a superior delivery vector through enhanced VEGF protein expression without compromising on cellular viability.

During wound healing, the process of vascularization is enabled by the activation of endothelial cells that contribute to the formation of a neo-vasculature.^26^ Similarly, dermal fibroblasts influence the behavior of endothelial cells and contribute to the structural support of new vessels through cell–cell signaling, paracrine support, extracellular matrix production and remodeling.^60^ Thus, providing a suitable environment for the promotion of this pro-regenerative cell–cell interaction is key for the successful healing of the tissue, and collagen-GAG scaffolds are already established as enabling platforms of this cellular cross-talk^53^ and the repair of wounds from non-chronic etiology, such as burn wounds.^61^ However, an additional stimulus is required to further drive healing in the intricate environment of chronic wounds. Our findings suggest that the inclusion of VEGF complexed GET nanoparticles into the collagen-GAG scaffolds provide a direct influence on this pro-angiogenic response obtained from dermal fibroblasts and endothelial cells in a 3D environment, with enhanced endothelial cell infiltration and organization into vascular network-like structures.

In contrast, incorporation of VEGF complexed lipofectamine nanoparticles on the scaffolds elicited a non-significant increase in VEGF expression from dermal fibroblasts, accompanied by a substantial decline in cell viability. Although endothelial cell viability remained unaffected within these scaffolds, no evidence of organized vascular-like structures was observed. These findings align with comparable outcomes reported in existing literature for both GET-based and lipofectamine-based delivery systems. Notably, Raftery *et al.*^43^ reported enhanced blood vessel formation and bone deposition without evidence of cytotoxicity when implanting VEGF and BMP-2-complexed GET-activated scaffolds in an *in vivo* rat calvarial critical-seized bone defect model, showcasing the therapeutic efficiency and safety of GET-based nucleic acid delivery. While the transfection efficiency and cytotoxic effects of lipofectamine are well-documented in 2D environments, their effect in 3D constructs remains less explored. Nevertheless, Monteforte *et al.*^62^ reported the delivery of lipofectamine-based nanoparticles from an alginate hydrogel to endothelial cells which enhanced the pro-angiogenic response at the expense of cell viability and proliferation, supporting the evidence that lipofectamine-based systems perform at the expense of cell viability. Collectively, these findings corroborate that GET-based nucleic acid delivery from VEGF-activated scaffolds provides a microenvironment conducive to angiogenesis including the production of pro-angiogenic growth factors, ensuring cell survival, and supporting cell organization.

The formation of neo-vasculature relies on migration and vascular network formation by endothelial cells, processes directed and supported by a number of pro-angiogenic factors, including VEGF.^27,63^ However, in a chronic wound environment, these growth factors are scarce, leading to a disruption in the natural sequence of wound repair.^63^ Dermal fibroblasts cultured on G-VEGF-activated scaffolds enhanced angiogenic paracrine activity, promoting both migration and vascular formation of endothelial cells critical for healing. In contrast, paracrine signaling from fibroblasts cultured on lipofectamine scaffolds led to a similar response in endothelial cell migration but no increase in the number of branch points, tube formation and total tube length. This reduced pro-angiogenic response with these scaffolds may be the result of lower VEGF expression and the presence of cell stress-associated molecules produced by transfection of fibroblasts using lipofectamine.^64^ Despite reduced VEGF expression, fibroblasts on L-VEGF scaffolds exhibited an enhanced expression of bFGF which may lead to improved endothelial cell migration comparable to that obtained in the GET-based group.^27,65–67^

In order to understand the 3D multicellular interactions pivotal to wound closure and enhanced healing, dermal fibroblasts and endothelial cells were co-cultured on the different scaffold groups without additional growth factor supplementation, consistent to the harsh growth conditions observed in a chronic wound environment. After 14 days, cell viability analysis and confocal imaging revealed the superior vascular organization of endothelial cells within the G-VEGF scaffolds, optimal for wound healing, contrasting with sparse endothelial cells observed in the gene-free and L-VEGF scaffolds. These observations align with the findings of Dohle *et al.*,^68^ who reported the evaluation of a porous fiber-hydrogel composite loaded with VEGF through an *in vitro* co-culture model with dermal fibroblasts and endothelial cells. Their study provided evidence of significantly enhanced microvessel formation exclusively in VEGF-containing composites, while VEGF-free composites exhibited smaller and decreased number of vascular-like structures. Building on these findings, we hypothesize that the heightened response with the G-VEGF-activated scaffolds stems from the combination of increased VEGF expression and low cytotoxicity, facilitating endothelial cell migration and organization into vascular-like structures. Conversely, we speculate that the lack of tube formation observed in the gene-free group may be attributed to the absence of early VEGF expression, leading to reduced endothelial cell activity and organization. Moreover, the cytotoxic effect from lipofectamine in the scaffolds results in cell death, leading to a lack of pro-angiogenic growth factors in the microenvironment that hamper the organization of endothelial cells and the overall reduced pro-angiogenic outcome.

Finally, given that nerve damage is a crucial but overlooked aspect of chronic wounds,^69^ the neurogenic potential of the scaffolds was studied. We demonstrated that the VEGF-activated scaffolds consistently enhanced neurite outgrowth in models of both motor and sensory neurons, showcasing the scaffold's potential to promote neurite outgrowth. In the case of growing neurons, the VEGF-activated scaffolds exhibited greater potential at maintaining the neurogenic phenotype of the cells as quantified by β-tubulin III expression, while enhancing neurite extension. Similarly, the culture of adult dorsal root ganglia sensory neurons on the VEGF-activated scaffolds showed a greater ability to promote neurite outgrowth. In particular, these findings provide clinically-relevant implications as chronic wounds are more common in older demographics where nerve regeneration is more challenging.

The upregulation of VEGF expression has been widely explored as a strategy to promote angiogenesis, particularly through topical treatments and biomaterial scaffolds. For instance, Wang *et al.* developed gold nanoparticles conjugated with *p*VEGF and the antimicrobial peptide LL37 for topical application in a diabetic mouse model, resulting in accelerated wound closure and increased CD31+ cell presence.^70^ Similarly, Lu *et al.* designed a hydrogel loaded with genetically modified, non-pathogenic bacteria to enhance VEGF expression in diabetic wounds, significantly improving closure rates.^71^ While these studies highlight the crucial role of VEGF in diabetic wound healing, topical treatments often suffer from limited targeting efficiency, and the use of non-native biomaterials or genetically modified organisms raises concerns for clinical translation. Given these challenges, we propose that localized *p*VEGF delivery from a biomimetic scaffold offers a promising alternative, potentially enhancing pro-angiogenic outcomes while improving clinical translatability. However, a multifunctional approach may still be required for optimal therapeutic efficacy.

We suggest that promoting an environment conducive to not only angiogenesis but also nerve regeneration may provide a solution for resolving chronic wounds, enhancing the recovery of sensation, and supporting healing.^6,7^ In the literature, several reports investigate the role and effects of VEGF in angiogenesis^70–73^ or nerve regeneration^74,75^ separately, but combined therapeutic approaches are less common. For instance, González-Pérez *et al.*^76^ developed an elastin-based hydrogel incorporating VEGF-mimetic (angiogenic) and laminin-derived (neurogenic) peptides. *In vivo* analysis in mice demonstrated increased directional formation of capillaries and long peripheral nerves at 6 weeks post-implantation, indicating the platform's potential for both vascularization and nerve repair. Although *in vivo* assessment is also needed to provide a clear comparison, we have shown the potential of the VEGF-activated scaffolds to promote both pro-angiogenic and pro-neurogenic outcomes through VEGF up-regulation in *in vitro* and *ex vivo* models, despite no additional neurogenic-specific stimulus.

Overall, these results showcase the successful development of a multifaceted VEGF-activated scaffold to promote pro-angiogenic and pro-neurogenic environments for chronic wound healing applications.

## 5. Conclusion

This study outlines the development of a multifaceted VEGF-activated scaffold platform for repair of chronic wounds by promoting pro-angiogenic and pro-neurogenic responses through VEGF up-regulation. The incorporation of VEGF complexed GET nanoparticles into a pro-regenerative collagen-GAG scaffold provides an enhanced therapeutic effect with lower cytotoxicity concern compared to commercially available transfection systems, underscoring its potential for safe and effective gene delivery. Moreover, the enhanced effect observed with the VEGF-activated scaffolds on crucial pro-angiogenic and pro-neurogenic processes, highlights the promising pro-regenerative potential of the scaffold for the treatment of chronic wounds.

## Ethics approval statement

The harvesting of dorsal root ganglia was approved by the RCSI Animal Research Ethics Committee (REC202005013) and under individual licence from the Health Products Regulatory Agency (HPRA, approval number AE19127/I259).

## Author contributions

JCP (data curation, formal analysis, investigation, methodology, and writing – original draft), MM (data curation, formal analysis, investigation, methodology, and writing – review and editing), COC (data curation, formal analysis, investigation, methodology, and writing – review and editing), AD (methodology and resources), JED (resources), CJK (conceptualization, supervision), SB (conceptualization, supervision, and writing – review and editing), FOB (conceptualization, supervision, funding acquisition, and writing – review and editing).

## Data availability

Data for this article are openly available at Open Science Framework (OSF) Repository at https://doi.org/10.17605/OSF.IO/7VARG under the terms of the Creative Commons Attribution 4.0 (CC-BY 4.0) license.

## Conflicts of interest

There are no conflicts to declare.

## Supplementary Material

BM-013-D4BM01051E-s001

BM-013-D4BM01051E-s002

BM-013-D4BM01051E-s003

BM-013-D4BM01051E-s004

BM-013-D4BM01051E-s005

BM-013-D4BM01051E-s006

BM-013-D4BM01051E-s007

BM-013-D4BM01051E-s008

BM-013-D4BM01051E-s009
