# Supplementary figures and images for: Development of a VEGF-activated scaffold with enhanced angiogenic and neurogenic properties for chronic wound healing applications
Source: Biomater Sci. 2025 Feb 14;13(8):1993–2011. doi: 10.1039/d4bm01051e (PMC11865941; doi:10.1039/d4bm01051e)

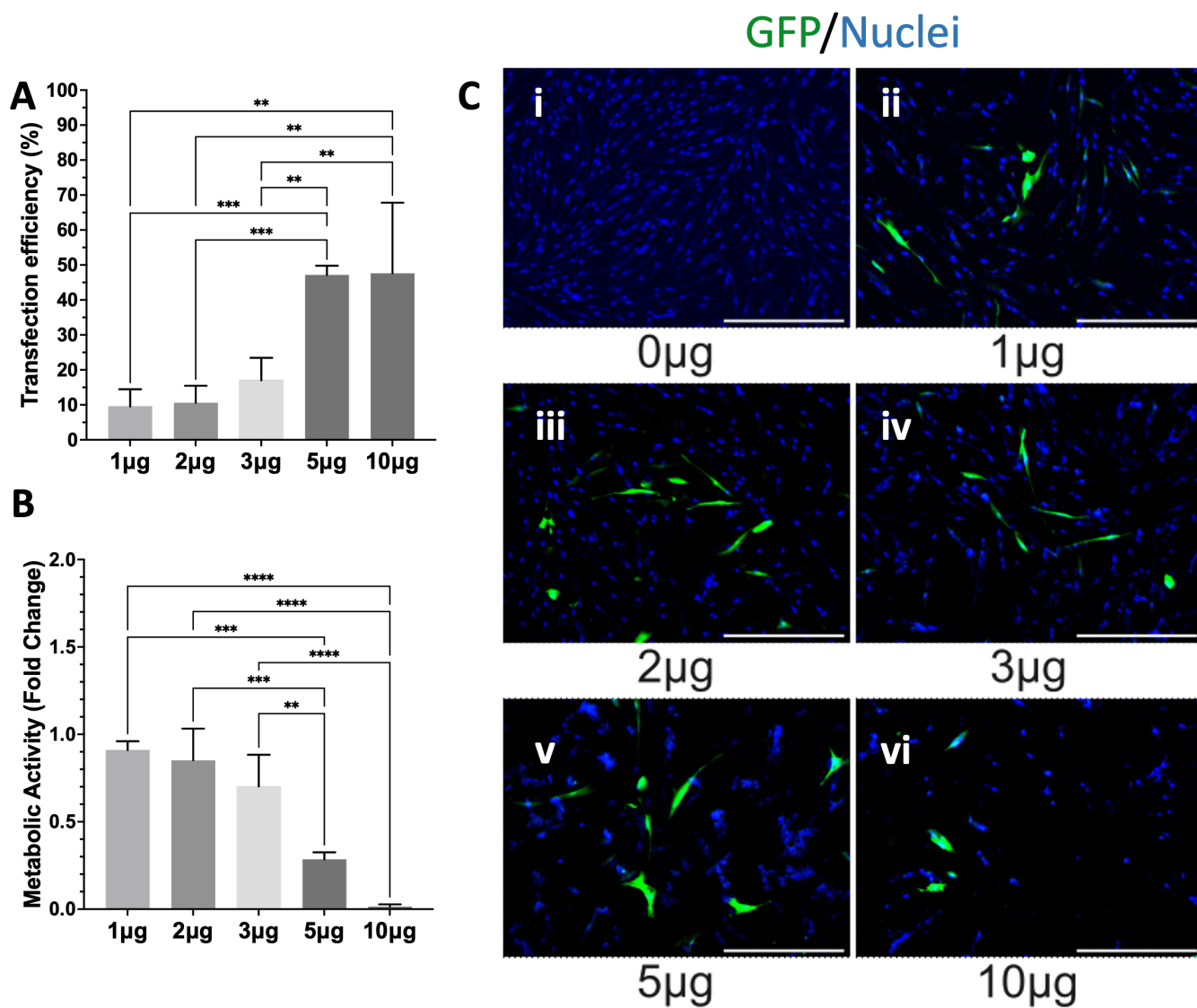

Supplement: BM-013-D4BM01051E-s001 [file BM-013-D4BM01051E-s001.pdf]

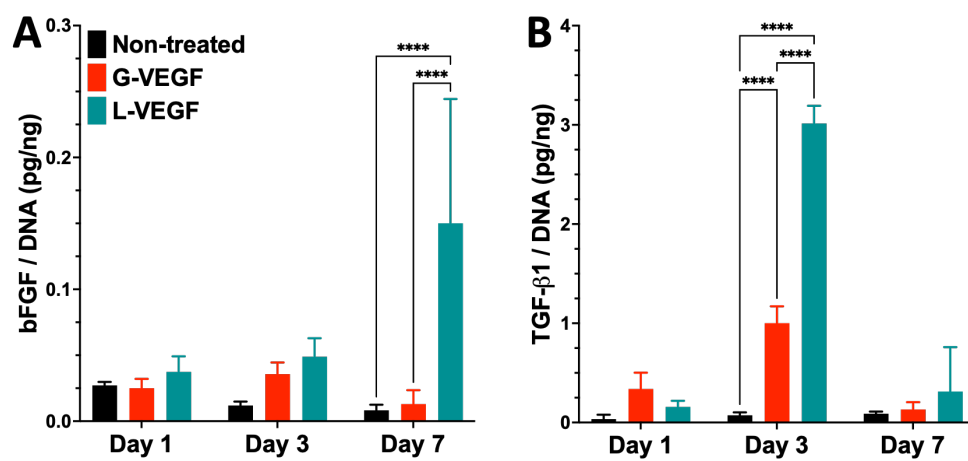

Supplement: BM-013-D4BM01051E-s002 [file BM-013-D4BM01051E-s002.pdf]

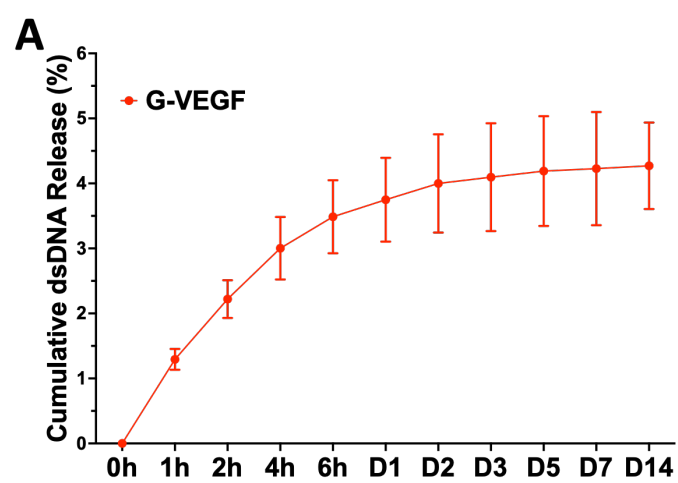

Supplement: BM-013-D4BM01051E-s003 [file BM-013-D4BM01051E-s003.pdf]

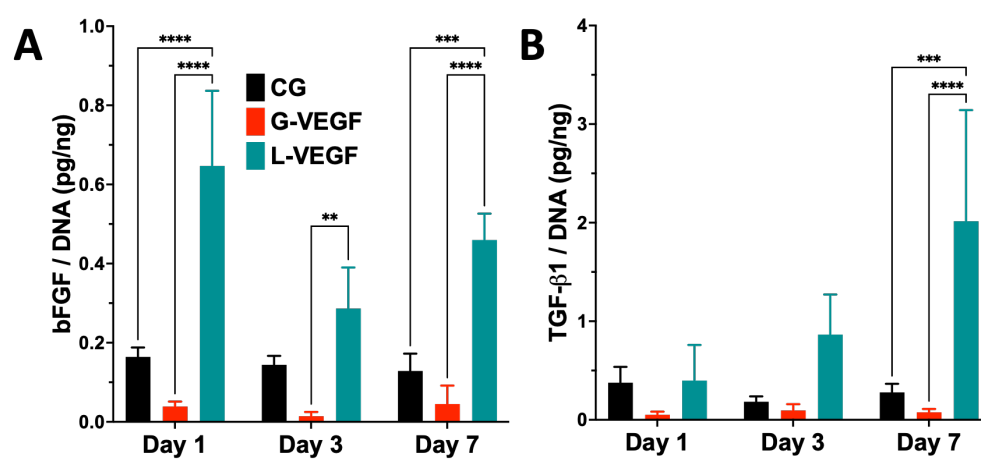

Supplement: BM-013-D4BM01051E-s004 [file BM-013-D4BM01051E-s004.pdf]

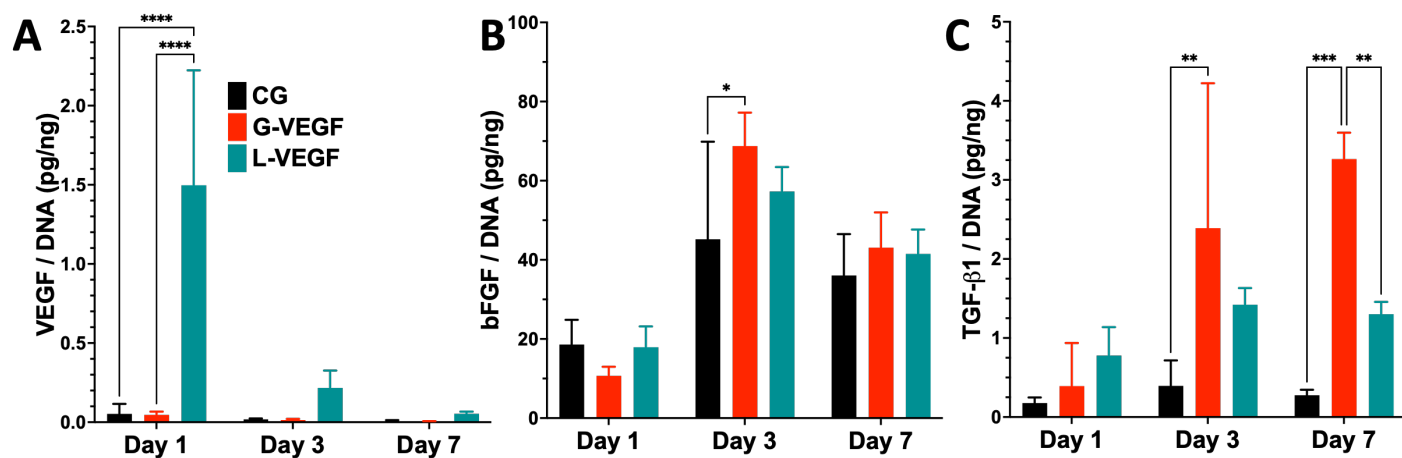

Supplement: BM-013-D4BM01051E-s005 [file BM-013-D4BM01051E-s005.pdf]

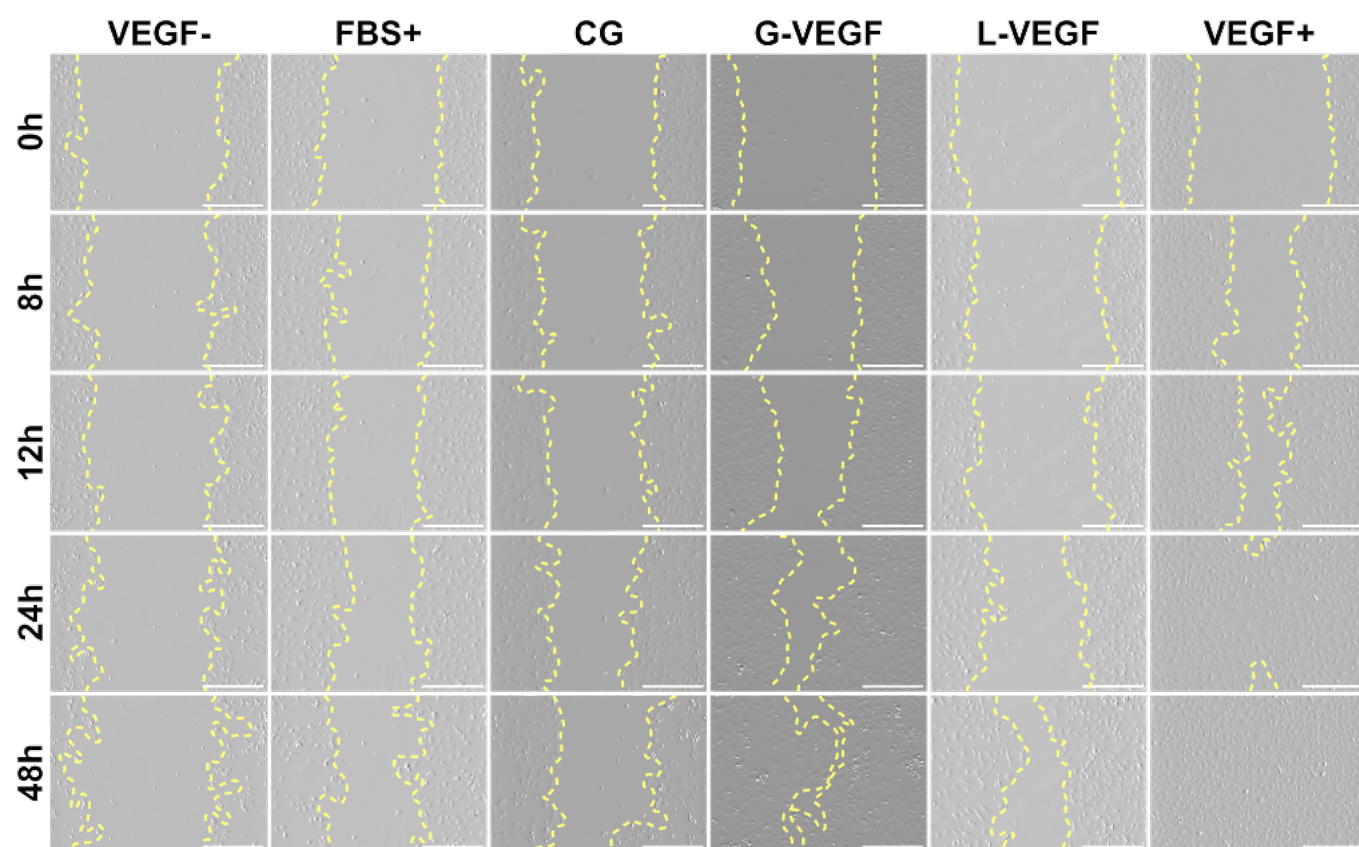

Supplement: BM-013-D4BM01051E-s006 [file BM-013-D4BM01051E-s006.pdf]

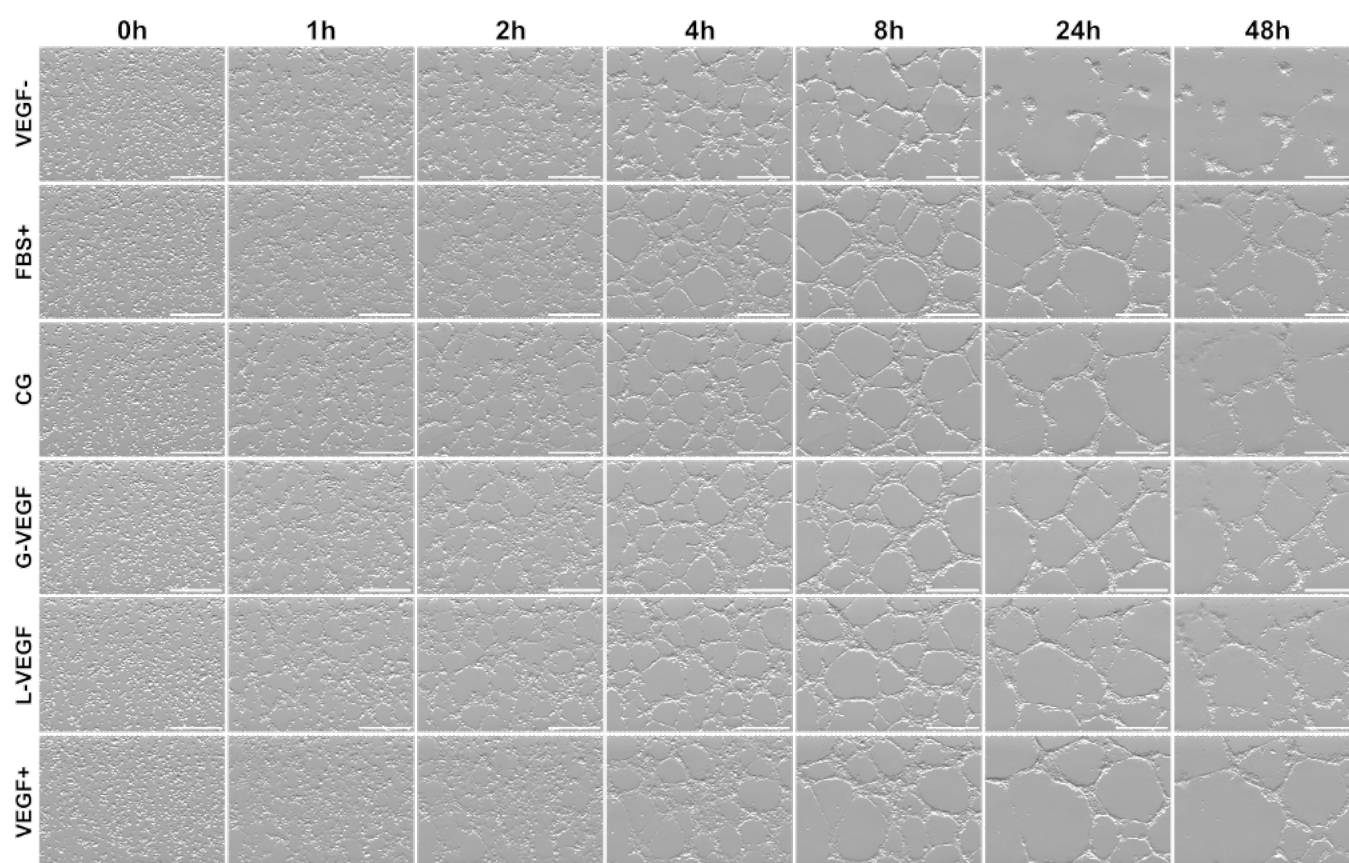

Supplement: BM-013-D4BM01051E-s007 [file BM-013-D4BM01051E-s007.pdf]

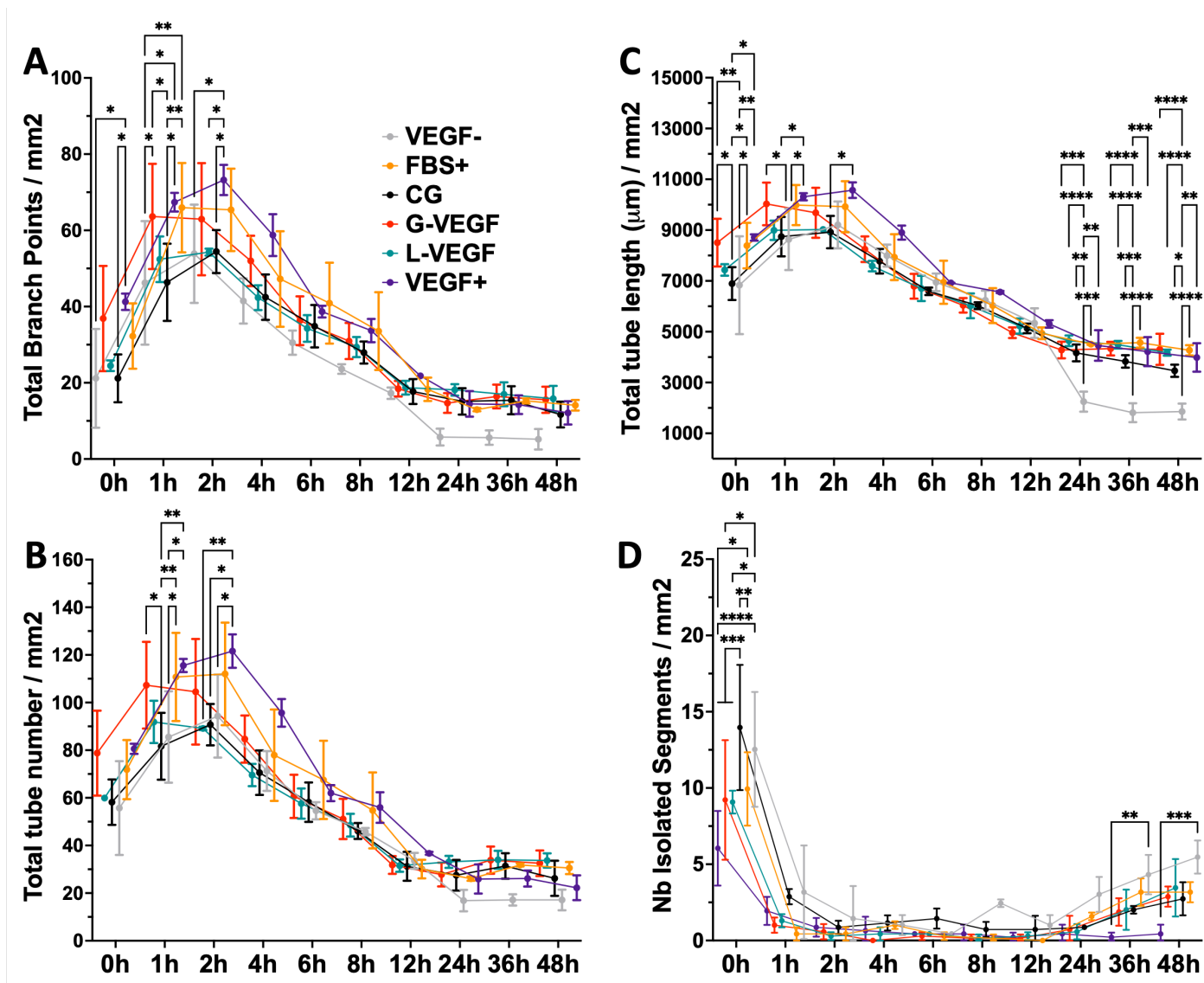

Supplement: BM-013-D4BM01051E-s008 [file BM-013-D4BM01051E-s008.pdf]
