# Supplementary material for: Development of a VEGF-activated scaffold with enhanced angiogenic and neurogenic properties for chronic wound healing applications
Source: Biomater Sci. 2025 Feb 14;13(8):1993–2011. doi: 10.1039/d4bm01051e (PMC11865941; doi:10.1039/d4bm01051e)
Supplement: BM-013-D4BM01051E-s009 [file BM-013-D4BM01051E-s009.pdf]

Supplementary Information (SI) for Biomaterials Science.
This journal is © The Royal Society of Chemistry 2025

Development of a VEGF-activated scaffold with enhanced angiogenic and neurogenic properties for chronic wound healing applications – Supplementary Information

*Juan Carlos Palomeque Chávez, Matthew McGrath, Cian O'Connor, Adrian Dervan, James E. Dixon, Cathal J. Kearney, Shane Browne, Fergal J. O'Brien**

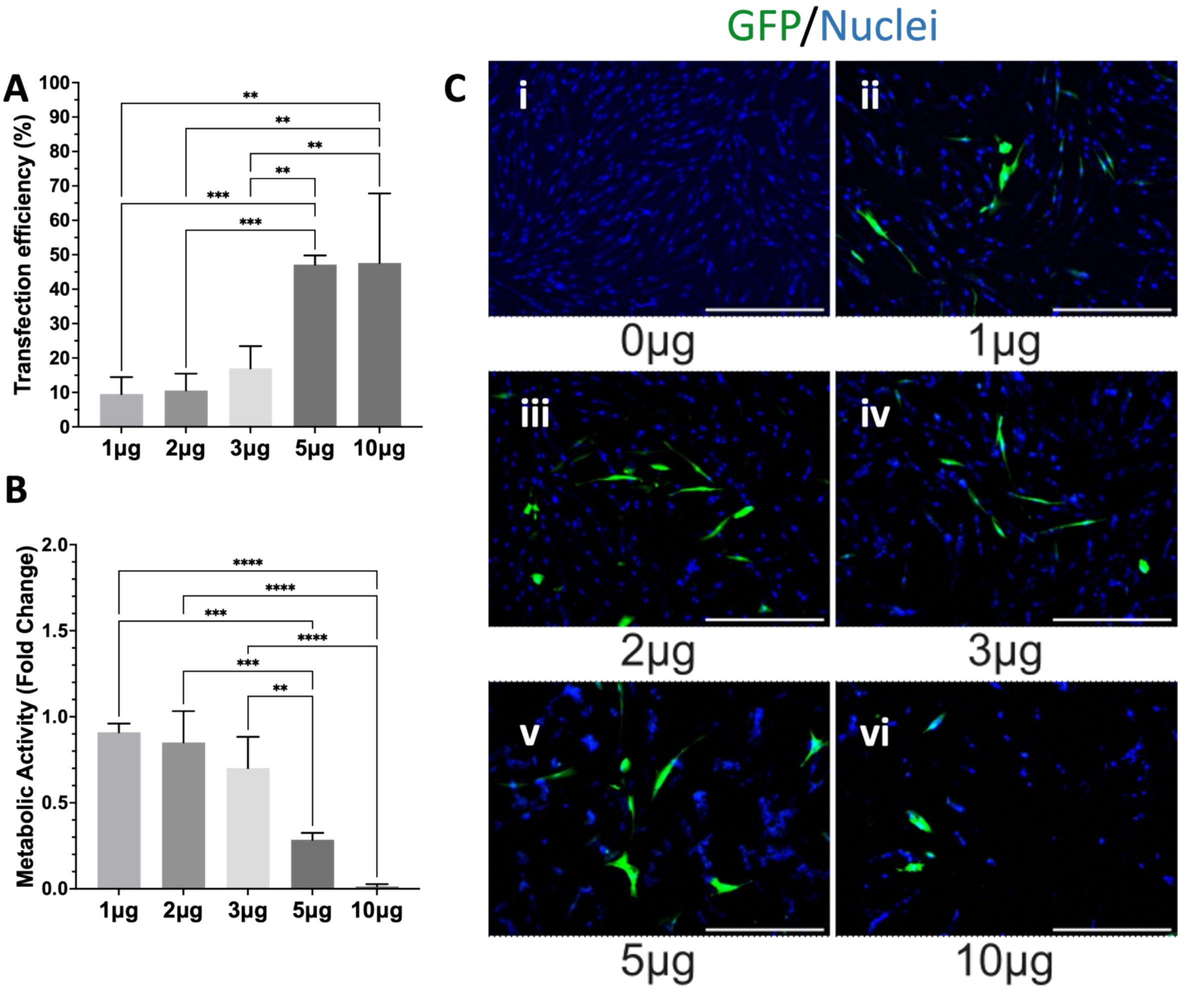

Supplementary Figure 1. Assessment of transfection efficiency (A), metabolic activity (B) and representative images of HDFs treated with different doses of G-GFP nanoparticles (C) used for the analysis of transfection efficiency. Dose displayed refers the amount of *p*DNA delivered per sample – Scale Bars = 500 μm. Data shows mean ± SD (n=3) and ** indicates $p < 0.01$, *** $p < 0.001$, **** indicates $p < 0.0001$.

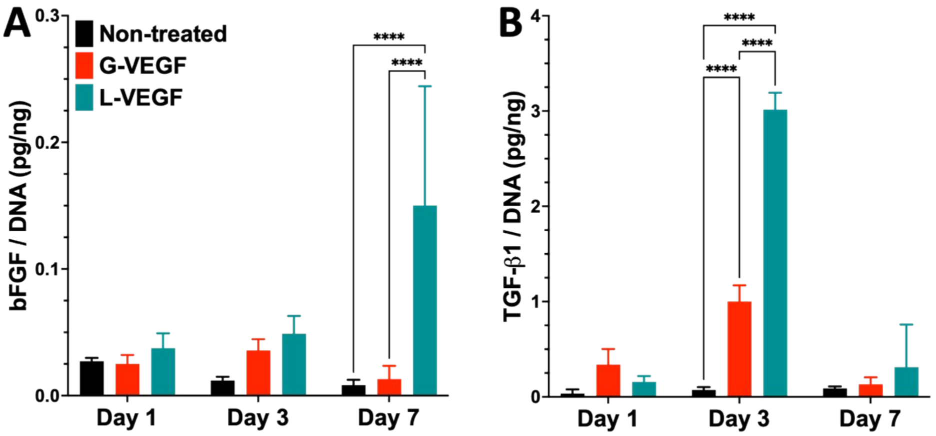

Supplementary Figure 2. Quantification of bFGF (A) and TGF-β1 (B) expression per unit of DNA in an *in vitro* monolayer of HDFs on days 1, 3 and 7 post-transfection. Data shows mean ± SD (n=3) and **** indicates $p < 0.0001$.

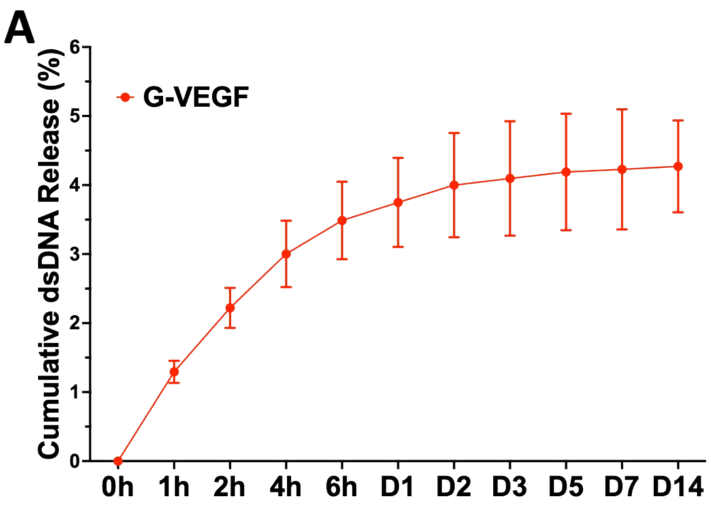

Supplementary Figure 3. Characterization of dsDNA release behavior from G-VEGF scaffolds over 14 days. Data shows mean ± SD (n=5) and **** indicates $p < 0.0001$.

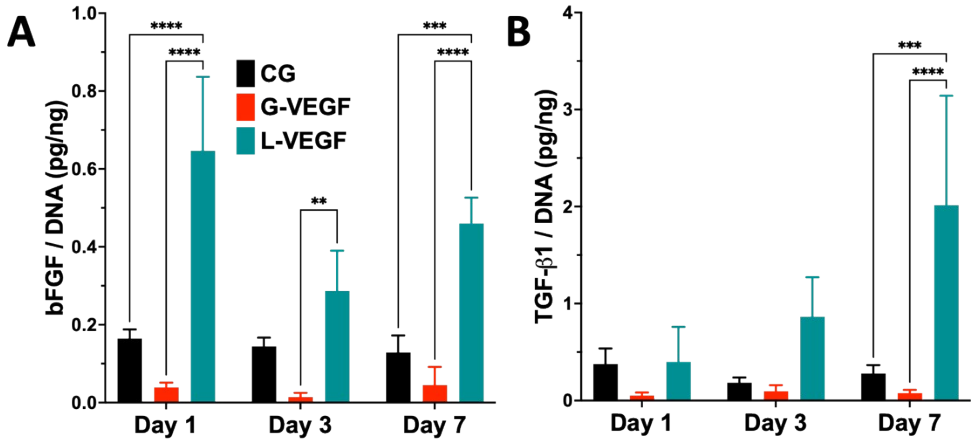

Supplementary Figure 4. Quantification of bFGF (A) and TGF-β1 (B) expression per unit of DNA of HDFs cultured on VEGF-activated scaffolds on days 1, 3 and 7 post-transfection. Data shows mean ± SD (n=3) and ** indicates $p < 0.01$, *** $p < 0.001$, **** $p < 0.0001$.

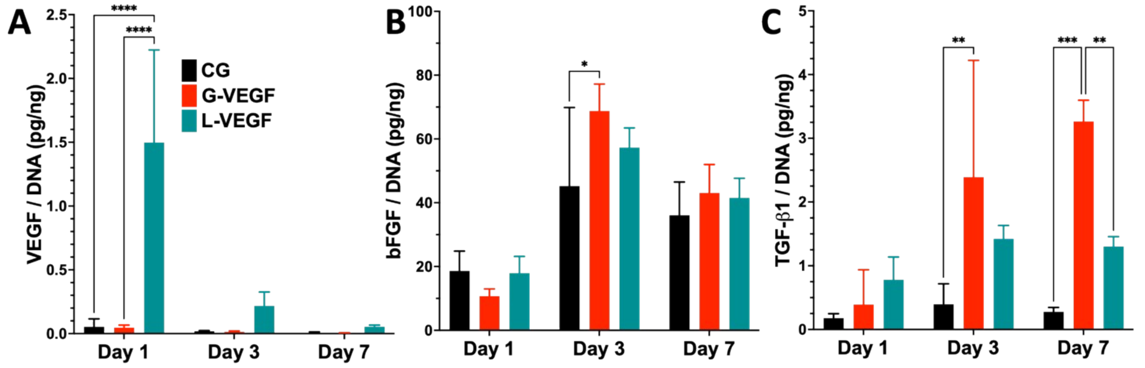

Supplementary Figure 5. Quantification of VEGF(A), bFGF (B) and TGF-β1 (C) expression per unit of DNA of HUVECs cultured on VEGF-activated scaffolds on days 1, 3 and 7 post-transfection. Data shows mean ± SD (n=3) and * indicates $p < 0.05$, ** $p < 0.01$, *** $p < 0.001$, **** $p < 0.0001$.

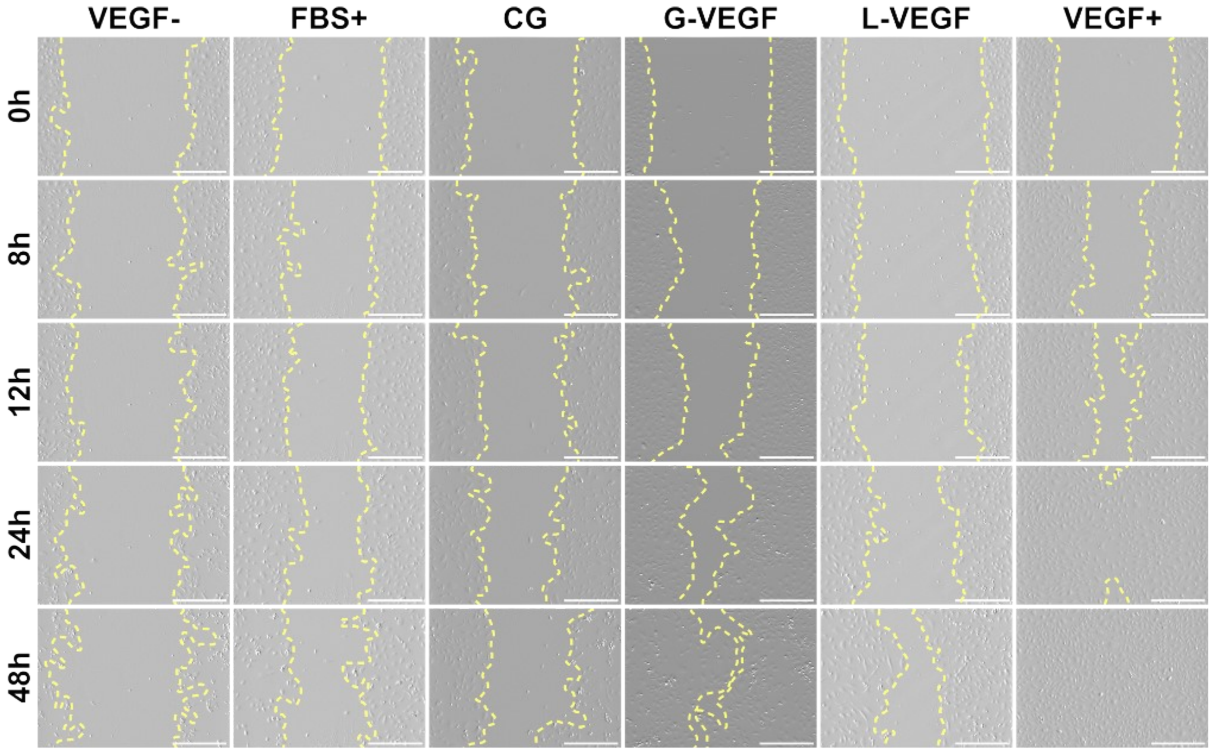

Supplementary Figure 6. Representative images of scratch assay carried out on HUVECs monolayers and treated with supernatant from HDFs cultured on VEGF-activated scaffolds. Scale bars = 500 µm

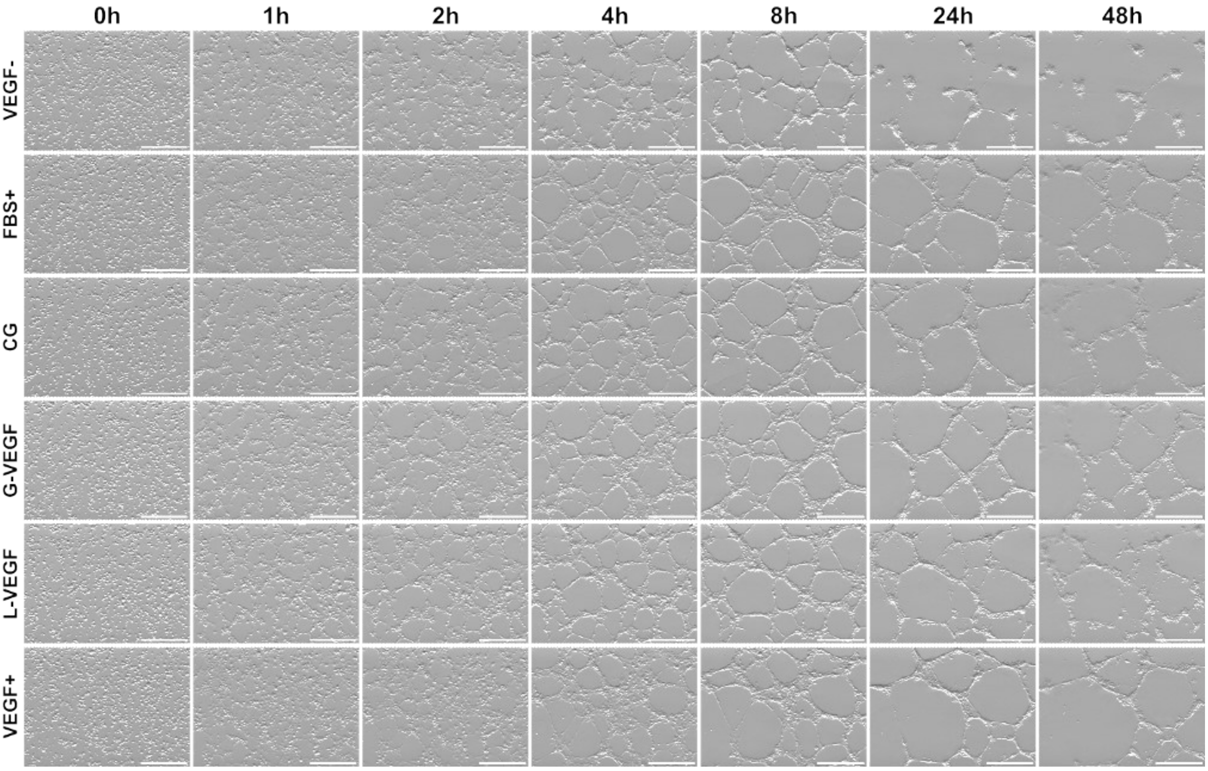

Supplementary Figure 7. Representative images of tube formation assay carried out with HUVECs treated with supernatant from HDFs cultured on VEGF-activated scaffolds. Scale bars = 500 μm

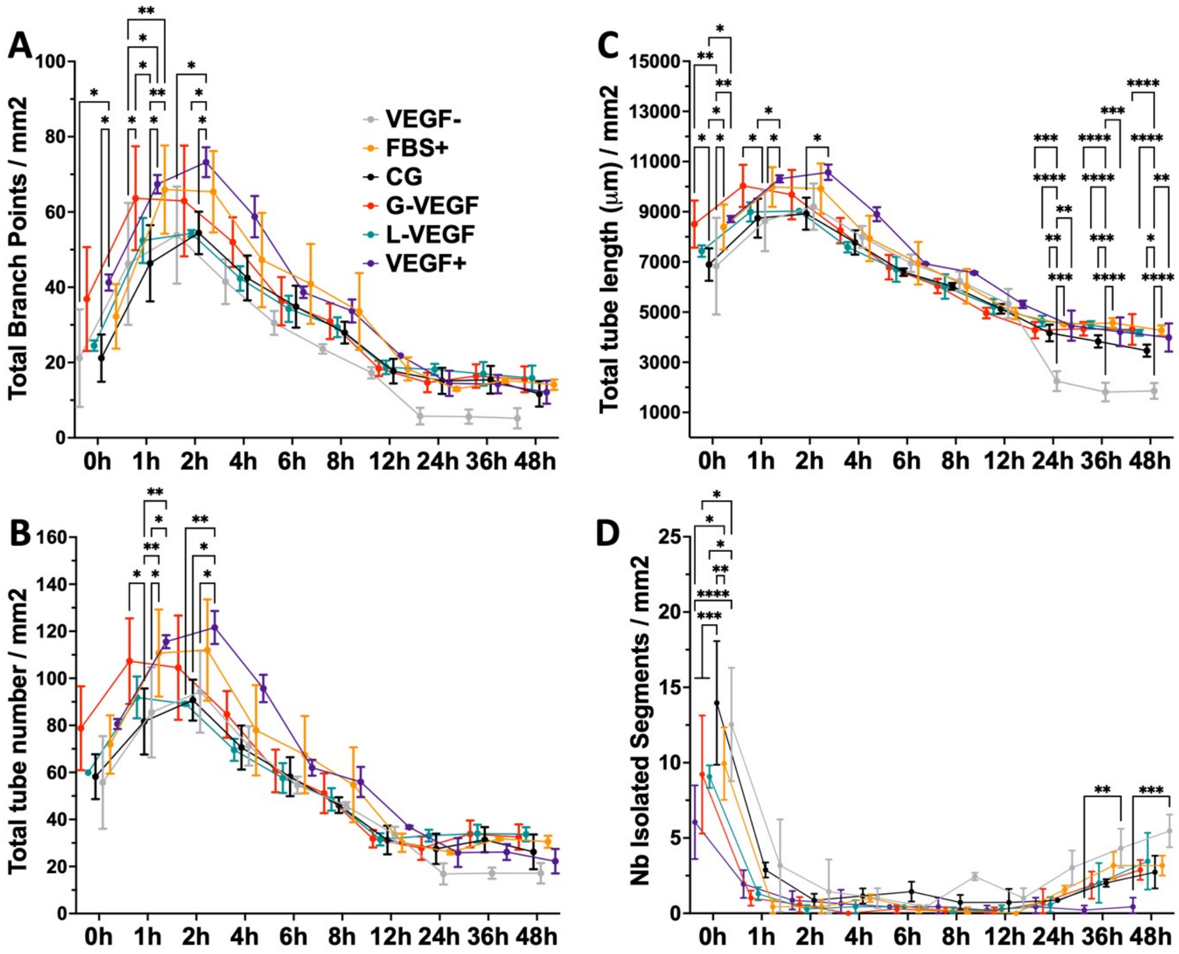

Supplementary Figure 8. Expanded analysis of the total number of branch points (A), total number of tubes (B), total tube length (C) and number of isolated segments (D) of HUVECs treated with supernatant from HDFs cultured on VEGF-activated scaffolds on day 3 post-transfection. Data shows mean ± SD (n=4) and * indicates $p < 0.05$, ** $p < 0.01$, *** $p < 0.001$, **** $p < 0.0001$.
